# Dynamic Multiscale Regulation of Perfusion Recovery in Experimental Peripheral Arterial Disease: A Mechanistic Computational Model

**DOI:** 10.1016/j.jacbts.2021.10.014

**Published:** 2022-01-05

**Authors:** Chen Zhao, Joshua L. Heuslein, Yu Zhang, Brian H. Annex, Aleksander S. Popel

**Affiliations:** a School of Pharmacy, Nanjing Medical University, Nanjing, Jiangsu, China; b Department of Biomedical Engineering, Johns Hopkins University School of Medicine, Baltimore, Maryland, USA; c Robert M. Berne Cardiovascular Research Center, University of Virginia, Charlottesville, Virginia, USA; d Department of Medicine, Medical College of Georgia, Augusta University, Augusta, Georgia, USA

**Keywords:** hindlimb ischemia, macrophage polarization, mathematical modeling, perfusion recovery, peripheral arterial disease, necrosis/necroptosis, systems biology, virtual mouse population, ARG1, arginase-1, EC, endothelial cell, HLI, hindlimb ischemia, HMGB1, high-mobility group box 1, HUVEC, human umbilical vein endothelial call, IFN, interferon, IL, interleukin, MLKL, mixed lineage kinase domain-like protein, PAD, peripheral arterial disease, RT-PCR, reverse transcriptase polymerase chain reaction, TLR4, Toll-like receptor 4, TNF, tumor necrosis factor, VEGF, vascular endothelial growth factor, VMP, virtual mouse population

## Abstract

In peripheral arterial disease (PAD), the degree of endogenous capacity to modulate revascularization of limb muscle is central to the management of leg ischemia. To characterize the multiscale and multicellular nature of revascularization in PAD, we have developed the first computational systems biology model that mechanistically incorporates intracellular, cellular, and tissue-level features critical for the dynamic reconstitution of perfusion after occlusion-induced ischemia. The computational model was specifically formulated for a preclinical animal model of PAD (mouse hindlimb ischemia [HLI]), and it has gone through multilevel model calibration and validation against a comprehensive set of experimental data so that it accurately captures the complex cellular signaling, cell–cell communication, and function during post-HLI perfusion recovery. As an example, our model simulations generated a highly detailed description of the time-dependent spectrum-like macrophage phenotypes in HLI, and through model sensitivity analysis we identified key cellular processes with potential therapeutic significance in the pathophysiology of PAD. Furthermore, we computationally evaluated the in vivo effects of different targeted interventions on post-HLI tissue perfusion recovery in a model-based, data-driven, virtual mouse population and experimentally confirmed the therapeutic effect of a novel model-predicted intervention in real HLI mice. This novel multiscale model opens up a new avenue to use integrative systems biology modeling to facilitate translational research in PAD.

## Highlights

• A first-of-a-kind systems biology computational model is presented that describes multiscale regulation of perfusion recovery in experimental peripheral arterial disease.
• Multilevel model calibration and validation enable high-resolution model simulations for experimental peripheral arterial disease (mouse HLI).
• An integrative model-based mechanistic characterization of the intracellular, cellular, and tissue-level features critical for the dynamic reconstitution of perfusion following different patterns of occlusion-induced ischemia in HLI is described.
• Using a model-based virtual HLI mouse population, pharmacologic inhibition of cell necrosis is predicted as a strategy with high therapeutic potential to improve perfusion recovery; in real HLI mice, the positive impact of this new strategy is then experimentally studied and confirmed.

## Visual Abstract

**Figure:**
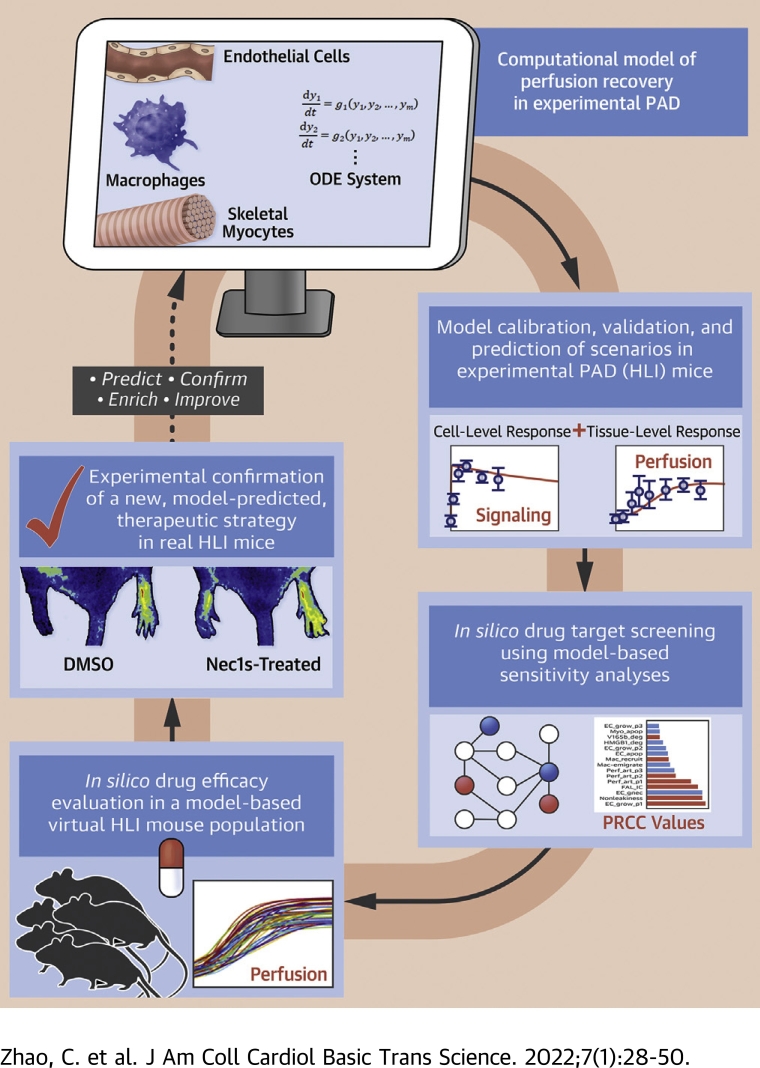

Peripheral arterial disease (PAD) affects >8 million people in the United States, and its prevalence increases significantly with age (1). PAD results from atherosclerosis, in which vascular occlusions in large leg arteries lead to varying degrees of ischemia in the distal lower limb skeletal muscle in patients, causing pain and inactivity. In severe PAD conditions (eg, critical limb ischemia), patients have rest pain and/or nonhealing ulcers or gangrene that can lead to amputation (2,3). Due to the significant occlusions in the major blood vessels that supply the legs, blood flow and perfusion to the lower limbs in patients with PAD become heavily dependent on the degree of vascular remodeling (from arteriogenesis and angiogenesis) in the ischemic limb muscle (4). Therapeutic agents and gene delivery aimed at stimulating vascular growth and remodeling in the ischemic limb by manipulating pro-angiogenic transcription factors (eg, hypoxia-inducible factor-1 alpha) or growth factors (eg, vascular endothelial growth factor [VEGF]) have, to date, largely failed (3,5). However, novel targeted strategies (eg, antibodies, macrophage- and stem cell-based therapies) are being actively investigated in preclinical studies as well as in early-phase clinical trials given the large unmet medical need (6, 7, 8, 9, 10).

In PAD, perfusion recovery in the ischemic muscle is a complex multiscale process involving a number of resident and mobilized cell types that dynamically participate in an orchestrated program of cellular signaling, communication, and tissue-level remodeling. For example, as a direct consequence of muscle ischemia, stressed and damaged skeletal myocytes and endothelial cells (ECs) can release massive amounts of high-mobility group box 1 (HMGB1), an early and endogenous danger signal that is suggested to broadly influence immune response, angiogenesis, and muscle regeneration (11,12). At the intracellular level, reduced blood supply is expected to lead to significant tissue ischemia and hypoxia that stabilize hypoxia-inducible factors within cells and activate the production of a number of strong pro-angiogenic factors (eg, VEGF, angiopoietins, matrix metalloproteinases) (13,14).

In addition to their direct pro-angiogenic effects on ECs, many of these factors regulate key processes in other cell types within the ischemic muscle. For example, VEGF has been shown to promote the survival and migration of myoblasts and their differentiation into skeletal myocytes, which are critical steps in postischemia skeletal muscle regeneration (15,16). Moreover, VEGF, by signaling through its receptor VEGFR1 on myeloid cells, can potently stimulate monocyte/macrophage recruitment, and this axis also contributes to the functional polarization of macrophages (eg, M1-like, M2-like) in the ischemic tissue microenvironment to modulate perfusion recovery (17, 18, 19). Macrophages, as shown by a number of studies, are quick responders following ischemic injury in PAD, and their infiltration and functional activities, such as their capacities to exacerbate or resolve inflammation and influence arteriogenesis and angiogenesis, are tightly controlled by the dynamic cytokine milieu and inflammatory status in the muscle (9,20, 21, 22). Compared with other immune cells, macrophages are present in relatively high quantities in the lower limb skeletal muscle (23,24), and they produce an array of biochemical signals during ischemia to communicate with other cells and themselves in paracrine and autocrine manners, in addition to performing phagocytosis. For example, pro-inflammatory M1-like macrophages secrete high levels of cytotoxic cytokines such as interferon (IFN)-γ and tumor necrosis factor (TNF)-α, which can potentially speed up apoptosis of ECs and skeletal myocytes (25, 26, 27). It was shown in the mouse model of PAD (hindlimb ischemia [HLI]) that M1-like macrophages dominate during the early phase of ischemic injury, whereas over time, this pro-inflammatory profile gradually diminishes as limb perfusion recovers, along with an increase in subjects’ walking ability (28).

VEGF molecules, including the alternatively spliced isoform VEGF_165_b, are also produced by macrophages in HLI mice and can differentially affect pro-angiogenic signaling on ECs (7,19). Furthermore, the functional polarization of macrophages is rarely all or none and is heavily influenced by autocrine and paracrine signaling (29). In addition to skeletal myocytes, ECs, and macrophages, many other cell types are found with varying abundance in the leg muscle of human and mouse (23,24); however, their cell dynamics and function in the pathophysiology of PAD remain much less understood and characterized.

Data from the accumulated literature on the individual components of myocyte–EC–macrophage signaling and communication in PAD have already projected a highly complex program that dynamically processes signals from multiple scales to regulate postischemic perfusion recovery. To systematically integrate these data into a dynamic analyzable biological framework and achieve a more complete understanding of the pathophysiological response and recovery of ischemic insult, we have developed the first computational model that mechanistically and quantitatively describes this multiscale (considering intracellular, cellular, and tissue-level processes) and multicellular (considering skeletal myocytes, ECs, and macrophages) regulatory program occurring in the skeletal muscle tissue in the context of PAD. We applied the model in 2 scenarios of experimental PAD (HLI induced by “acute” vs “gradual” procedures) to describe the distinct recovery response in different modes of limb ischemia (30). We then computationally calibrated and validated the model using multilevel, quantitative time-course literature data on pathway activation and intracellular signal transduction, cytokine-mediated cell-level response, and tissue-level perfusion. In addition, a number of reported physiological features and preclinical observations with important translational values in PAD, such as the changes in perfusion recovery in HLI after the administration of different targeted therapeutics, were taken into account by our model.

Using this model, we characterized the dynamic and continuous polarization spectrum in macrophages infiltrating the tissue after HLI, with a very high quantitative and temporal resolution of their signaling and phenotype marker expression. Furthermore, we screened and tested several novel therapeutic strategies as proposed by our model sensitivity analysis in silico in a virtual mouse population with HLI and evaluated their relative efficacies in terms of improvement in perfusion recovery and muscle regeneration. Finally, we experimentally validated the model by showing that inhibition of cell necrosis in HLI (as a top-ranked therapeutic intervention predicted by our computational model) can indeed improve perfusion recovery in HLI mice. In short, the multiscale computational model presented here, as the first endeavor to use a mechanism-based systems biology framework to simulate and describe PAD pathophysiology in preclinical settings, can be a useful in silico platform that provides novel systems-level insights and rich mechanistic information with high temporal and quantitative resolution for the future translational investigation of PAD and drug development.

## Methods

### Mice

All animal protocols were approved by the Institutional Animal Care and Use Committee at the University of Virginia (Protocol 3721) and conformed to all regulations for animal use outlined in the American Heart Association Guidelines for the Use of Animals in Research. C57BL/6 mice and BALB/c mice were purchased from Charles River Laboratory. All animals were housed in the animal facilities at the University of Virginia.

### Murine model of HLI

We used a previously detailed HLI scheme (7,9,31). Before surgery, male mice, 10 to 12 weeks of age, were anesthetized with 1.5% to 2% isoflurane, and the lower limbs were shaved, depilated to remove excess hair, and prepped for aseptic surgery. On the day of surgery, mice were anesthetized (120 mg/kg ketamine and 10 mg/kg xylazine intraperitoneally). Ophthalmic ointment was applied to both eyes according to ointment instructions. With the animal in the supine position, a longitudinal incision approximately 1.5 cm long was made along the left medial thigh beginning at the inguinal ligament. The femoral artery was first ligated by using 8-0 Prolene sutures (Ethicon), then resected from just above the inguinal ligament to its bifurcation at the origin of the saphenous and popliteal arteries proximal to the saphenous artery bifurcation. The surgical site was then closed with 5.0 Prolene sutures. A sham surgery, wherein the femoral artery was exposed but not ligated, was performed on the contralateral hindlimb (eg, right leg).

Animals received subcutaneous injections of buprenorphine for analgesia immediately after surgery and every 12 hours for the next 48 hours. In addition, all mice received prophylactic treatment with antibiotics (Baytril, Bayer Corporation) and analgesics (2 mg/mL acetaminophen) in their drinking water for 7 days after surgery. BALB/c mice were treated with either 1.6 mg/kg/d Nec-1s or vehicle control (0.05% dimethyl sulfoxide) intraperitoneally for 7 days after HLI surgery.

Only male mice were used in the current study. There was no active process of selection of one sex or another. A short list of manuscripts from the Annex laboratory will show that either sex has been used in similar studies (31, 32, 33, 34, 35). Therefore, to avoid the need to stratify mice based on sex and to avoid the unnecessary use of animals, the required number of mice of one sex were used.

### Laser doppler perfusion imaging and necrosis scoring

For monitoring blood flow recovery and postsurgical ischemia, mice were anesthetized via 1.5% isoflurane under constant oxygen. Mice were placed in a prone position, and the soles of the feet were scanned (PeriCam PSI, PeriMed). Mean foot perfusion was used to calculate relative perfusion ratio (ligated/unligated).

A necrosis grading system was used to evaluate post-HLI limb necrosis, as done previously (31,32,36). The system included the following: grade 1, involving only the toes; grade 2, extending to the dorsum pedis (top of foot); grade 3, extending to the metatarsal plantar (bottom) skin surface; and grade 4, extending past the ankle into the thigh or complete limb necrosis. Any mice with a score ≥3 were euthanized according to humane endpoints.

### Tissue harvesting and RNA isolation

Seven days after HLI surgery, mice were anesthetized (120 mg/kg ketamine and 10 mg/kg xylazine intraperitoneally) and euthanized via an overdose of carbon dioxide exposure, confirmed by cervical dislocation. Both gastrocnemius muscles were dissected free, cut in half, and processed for protein expression and/or RNA isolation.

For total RNA isolation, tissues were incubated in 450 μL of TRIzol reagent (Thermo Fisher Scientific) for 5 minutes at room temperature. Tissues were then placed on ice and homogenized by using a power homogenizer (Omni International) in short bursts to avoid overheating. After homogenization, an additional 550 μL TRIzol reagent was added. Samples were incubated for another 5 minutes at room temperature to ensure complete lysis. A total of 200 μL of chloroform was added to each sample. Samples were then shaken vigorously for 15 seconds and incubated for 3 minutes at room temperature. After this incubation, samples were centrifuged for 10 minutes at 12,000 *g* at 4°C. The resulting aqueous layer was carefully removed, place in a new RNA/DNase-free tube, and an equal volume of 70% ethanol was added to the saved aqueous layer. RNA isolation then proceeded using the PureLink total RNA purification system (Life Technologies Inc, catalog no. 12183025) with the on-column DNase protocol (Life Technologies Inc) according to the manufacturer’s instructions. RNA concentration and purity were determined with a NanoDrop spectrophotometer (Thermo Fisher Scientific) in duplicate.

### Quantitative reverse transcriptase polymerase chain reaction

For quantitative reverse transcriptase polymerase chain reaction (RT-PCR), 600 ng of total RNA was reverse transcribed by using the SuperScript III First-Strand Synthesis SuperMix (Thermo Fisher Scientific, #11752-05) according to the manufacturer’s instructions. After reverse transcription, RT-PCR was performed on 30 ng complementary DNA using SensiMix II Probe (Bioline, BIO-83005) and TaqMan Primer Assays (Thermo Fisher Scientific) for HPRT-VIC, RIP1K, RIP3K, and mixed lineage kinase domain-like protein (MLKL) on a CFX96 Real Time Detection System (Bio-Rad). Normalized expression to HPRT was quantified by using the comparative 2^ΔΔCt^ method.

### Western blot

Dissected gastrocnemius tissues were placed in 1 mL ice-cold radioimmunoprecipitation assay buffer supplemented with protease inhibitor (MilliporeSigma, 1:100, #P8340) and homogenized on ice by using a power homogenizer (Omni International) in short bursts to avoid overheating. Lysed samples were then cleared for 30 minutes at 4^°^C under constant agitation. Samples were centrifuged for 1 minute at 10,000 *g*, the supernatant was collected, and a Pierce BCA assay (Thermo Fisher Scientific, #23225) was used to determine total protein concentration. Samples were diluted 1:1 in 4× XT sample buffer (Bio-Rad, #161-0791) with XT reducing reagent (Bio-Rad, #161-0792, 1× final concentration), and boiled for 10 minutes. Equal protein was loaded onto a 4% to 12% Bis-Tris Criterion XT pre-cast gel (Bio-Rad, #3450123) and run at constant voltage in XT MOPS running buffer (Bio-Rad, #161-0791). Protein was then transferred for 1 hour at 1 amp constant current onto a nitrocellulose membrane.

After transfer, membranes were stained with Ponceau S for 15 minutes at room temperature to determine total protein. Blots were then de-stained in phosphate-buffered saline and blocked for 1 hour at room temperature in Tris-buffered saline with Tween 20 (TBST) + 5% bovine serum albumin and then incubated with primary antibodies overnight at 4^°^C. Western blots were performed by using primary antibodies directed against MLKL. After overnight incubation, blots were washed 5× for 5 minutes in TBST before being incubated in secondary antibody for 1 hour at room temperature. Goat anti-rabbit horseradish peroxidase–conjugated secondary antibodies were purchased from Cell Signaling Technology (#7074) and used at a 1:5,000 dilution. Blots were again washed 5× in TBST. Bands were then developed by using Clarity Western ECL Substrate (Bio-Rad, #170-5061) followed by detection using a ChemiDoc Touch Imaging System (Bio-Rad). Images were quantified by using the Image Studio Lite software program (LICOR). Total MLKL expression was normalized to total protein as determined by Ponceau S staining.

### Cell culture

Human umbilical vein ECs (HUVECs) were purchased from Cell Applications and used between passages 3 and 6. HUVECs were cultured in All-In-One Endothelial Growth Medium (Cell Applications, catalog no. 211-500). Upon reaching confluence, media were changed to endothelial starvation media (Cell Applications, catalog no. 209-250) and cells were incubated in a 2% oxygen chamber (BioSpherix) for 0, 24, or 48 hours. HUVECs were directly lysed in TRIzol, and total RNA isolation then proceeded using the PureLink total RNA purification system with the on-column DNase protocol according to the manufacturer’s instructions. RNA concentration and purity were determined with a NanoDrop spectrophotometer in duplicate.

### Human patient population

All protocols were approved by the institutional review boards at East Carolina University and the University of Virginia. Complete patient demographic characteristics have been published previously (37). Total RNA from limb biopsy samples was reverse transcribed by using the SuperScript III First-Strand Synthesis SuperMix (#11752-05) according to the manufacturer’s instructions. After reverse transcription, RT-PCR was performed on 7.5 ng complementary DNA using SensiMix II Probe (BIO-83005) and TaqMan Primer Assays for ACTB-VIC and MLKL on a CFX96 Real Time Detection System (Bio-Rad). Relative normalized expression to HPRT was quantified by using the comparative 2^ΔΔCt^ method and was tested for statistical significance by using Student’s t-test.

### Multiscale model scope and formulation

**Figure 1:**
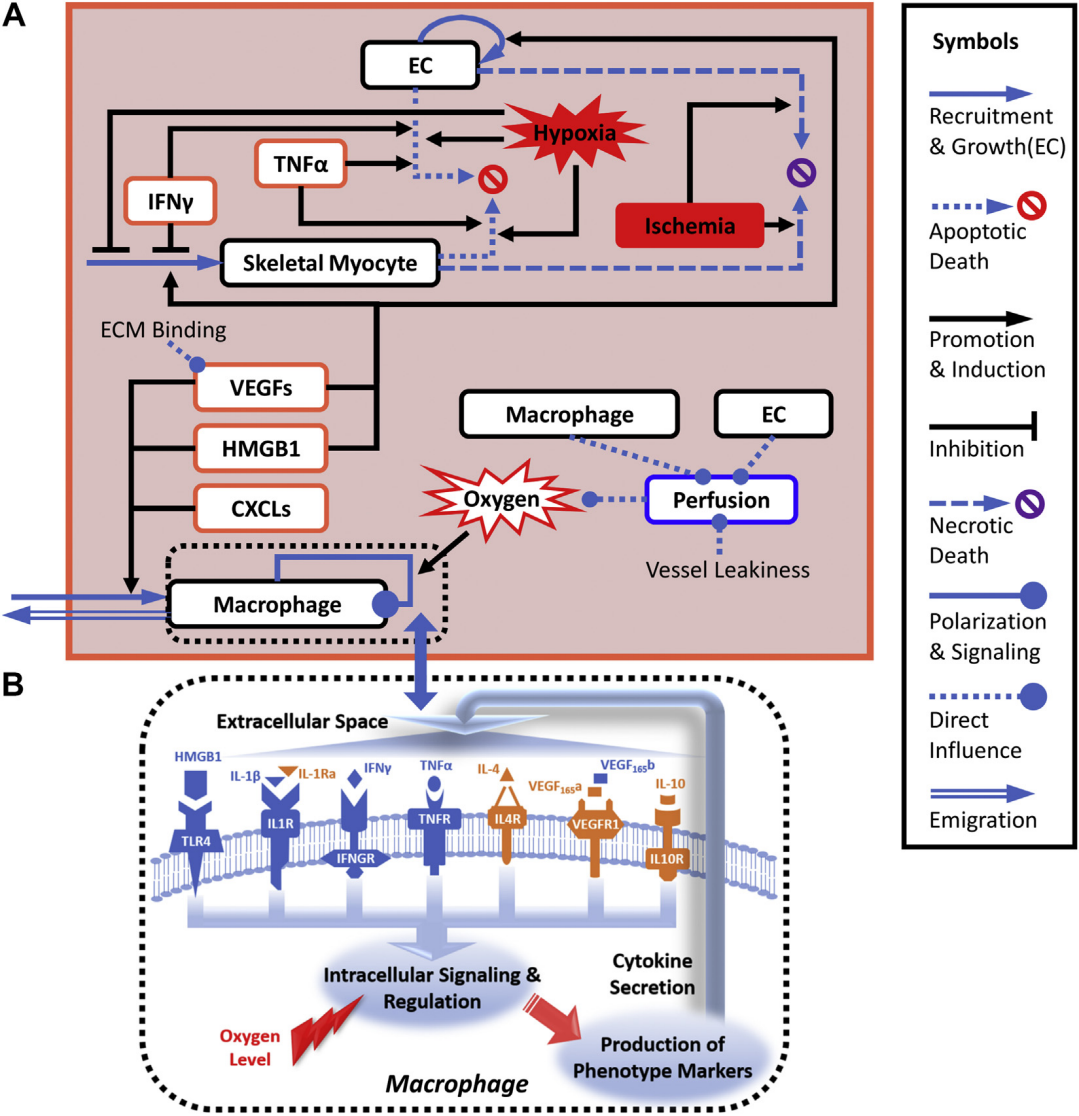
Overview of the Multiscale Computational Model **(A)** At the tissue level, mechanistic interactions (eg, through cell-secreted cytokines, shown in **orange rectangles**) between 3 representative cell types (myocytes, endothelial cells [ECs], and macrophages, shown in **black rectangles**) in the skeletal muscle tissue during hindlimb ischemia (HLI) were described **(symbols given on the right)**. In brief, the recruitment/growth and death/removal of these cells are differentially regulated by different cytokines and ischemia. A full description of all model interactions is available in Supplemental Table 1. **(B)** Among the 3 cell types, macrophage populations, given their high heterogeneity and plasticity, were explicitly represented by instances created from a cell-level multipathway mechanistic model that interfaces with other tissue-level components. The final macroscopic tissue perfusion level after HLI is dynamically influenced by both cellular (eg, population size of ECs/macrophages) and noncellular factors (eg, vascular leakage). CXCLs = CXC chemokine ligands; HMGB1 = high-mobility group box 1; IFN = interferon; IFNGR = interferon gamma receptor; IL = interleukin; TLR4 = Toll-like receptor 4; TNF = tumor necrosis factor; VEGF = vascular endothelial growth factor.

In our multiscale computational model, as shown in Figure 1, we explicitly included 3 cell types (skeletal myocytes, ECs, and macrophages) to describe limb tissue ischemia (eg, HLI), and each cell type is assumed to have one coarse-grained production (eg, recruitment, differentiation, and proliferation steps are merged accordingly depending on the specific cell type) and one constitutive removal rate (eg, apoptosis, emigration), both of which can be dependent on tissue oxygen and cytokine levels. In addition, we assumed that skeletal myocytes and ECs will undergo necrotic cell death during severe tissue ischemia at a rate proportional to the level of perfusion deficit (30,38). The quantitative relationships between cytokine or hypoxia levels and rates of cellular processes modeled (eg, dose–response relationship between TNF-α concentration and EC apoptosis) were estimated, mostly in the form of Hill-type equations, based on curated literature data (summarized in detail in Supplemental Table 1). For the macrophage component, we expanded a published multipathway cell-level computational model of macrophage polarization (29) by adding a new HMGB1/Toll-like receptor 4 (TLR4) axis (summarized in Supplemental Table 2) given the importance of HMGB1 in HLI and its impact on macrophage signaling. The new 8-pathway cell-level macrophage model was then fully incorporated into our multiscale model to enable a comprehensive description of the differential temporal activation of heterogeneous macrophage populations.

For cytokines, secretion of HMGB1 and VEGF isoforms (VEGF_165_a and VEGF_165_b) from myocytes and ECs was modeled with rates estimated from literature measurements. HMGB1 was secreted by necrotic but not apoptotic cells in high amounts in our model (39). The model further assumed that pro-inflammatory cytokines such as IFN-γ and TNF-α are only actively produced by macrophages but not the other 2 cell types, according to data from the literature (40,41). The high-level tissue perfusion is semi-mechanistically modeled as a variable that is constantly regulated based on the sum of 2 dynamic processes: the formation of new blood vessels, as represented by net EC growth, and by extent of arteriogenesis, which is dependent on the degree of occlusion-induced shear stress changes and macrophage infiltration (42,43). In the model, we also included the impact of several biological factors of high importance in the perfusion recovery in PAD, such as the leakiness of the newly formed blood vessels and the degree of VEGF sequestration by extracellular matrix (44,45).

In summary, our model comprises 2 primary components: a cell-level macrophage signaling component that depicts complex intracellular signal transduction and a tissue-level component. Both components were formulated based on ordinary differential equations: the cell-level macrophage component was built upon a published model (29) by introducing a new pathway (consisting of 4 new species and 8 new reaction mechanisms). The tissue-level component was constructed de novo, and it includes a total number of 34 species and 23 reactions and rules. Details regarding all reaction descriptions, equations, parameter values, and initial conditions of the cell- and tissue-level components are summarized in Supplemental Protocol 1 and Supplemental Tables 1 and 2. All model reactions, rules, and data in both components were compiled in the MATLAB SimBiology Toolbox (MathWorks); all model simulations (combining the cell- and tissue-level components) were executed by using MATLAB scripts, and the ode15s solver in MATLAB was used.

For calibration of the newly added HMGB1/TLR-4 axis in cell-level macrophage component, a global optimization algorithm in MATLAB (*patternsearch*) was used. For that, we used the same unit conversion method as described in a previous study (46) to compute the corresponding molecular initial conditions for the model to simulate the different HMGB1 concentrations used in in vitro experiments. For the tissue-level component, details regarding the estimation of parameters, selection of initial conditions, and calibration are described in Supplemental Protocol 1 and Supplemental Table 1. ImageJ software (National Institutes of Health) was used for the blot densitometry analysis and other image measurements during quantification of experimental data. To ensure reproducibility, model SBML code (in .xml format) and a MATLAB script to run the model and generate sample simulations are available at https://github.com/czhaoqsp/padmodel.

### Model simulation and analysis settings

To mechanistically interface the cell-level heterogeneity with tissue-level activities under the deterministic model setup, the entire simulation time span (in weeks) was divided into smaller discrete intervals, which are in the unit of hours (up to 24 hours/1 day). At the beginning of each interval, the predefined initial conditions were used to simulate all the cell-level macrophage model instances along with the tissue-level component in parallel, and the resulting values (at the end of the current interval) from the macrophage model instances as well as the tissue-level component were used to compute the corresponding new initial conditions for the immediate next interval. For results of macrophage phenotype simulations, we used 4 hours as the simulation interval; all other simulations were generated using 24 hours as the interval duration. Time-course model behaviors in the 4-hour simulation runs are highly similar to those in the 24-hour runs. Details regarding this cell–tissue interface and initial condition computing process are available in Supplemental Protocol 1 and the sample MATLAB script that we provided.

The 2 scenarios of HLI (acute vs gradual) were represented in the model by setting reductions in the perfusion levels in 2 different ways; the degrees of reductions were calculated based on corresponding in vivo mouse data from literature. To simulate acute HLI, we assumed that the surgery was performed before day 1 (eg, on day 0); thus, this is reflected as a single drop in the perfusion day 1 initial condition (which is part of the initial conditions for the very first simulation interval). To simulate gradual HLI, a numerical time-dependent decrease (computed by using a fitted exponential) is applied to the initial conditions of perfusion at the beginning of all intervals during the entire simulated time span. More details about this procedure are described in Supplemental Protocol 1. A summary of literature data used in model calibration and validation can be found in Supplemental Table 3.

Methodologic details of other model analyses presented in this paper such as generation of a virtual mouse population (VMP) and simulation of targeted interventions are available in Supplemental Protocol 1.

### Definition of macrophage M1/M2-like scores

For the analysis of macrophage phenotypes, we introduced an integrative non-dichotomous metric “M1/M2-like score,” as done in several previous studies (29,46,47). In our model, this score is the division of the product of eight M1-like markers (TNF-α, IFN-γ, inducible nitric oxide synthase, interleukin [IL]-1β, IL-12, CXC chemokine ligand 9, CXC chemokine ligand 10, and VEGF_165_b) by the product of five M2-like markers (IL-10, IL-1Ra, VEGF_165_a, IL-4, and arginase-1 [ARG1]) to quantify the relative overall dominance of M1-like (eg, pro-inflammatory) versus M2-like (eg, anti-inflammatory) phenotypes in macrophages. For analysis and display, the calculated M1/M2-like scores under different conditions were then normalized to the baseline value (at the control condition for macrophages) and then log_10_ transformed. Thus, the relative macrophage M1/M2-like scores will take on numerical values from a wide continuous range, and a larger value would indicate an overall more pro-inflammatory M1-like phenotype (and vice versa).

### Model sensitivity and statistical analyses

Model sensitivity analyses at the tissue and cell levels were performed by using the partial rank correlation coefficient algorithm (and code) as described in Marino et al (48). More technical details are provided in Supplemental Protocol 1.

Statistical analysis for model-based comparisons in the VMP was performed in MATLAB, and a 2-tailed paired Student’s t-test was used. Statistical significance was set at *P* < 0.05. Data are presented as mean ± SEM. In the experimental investigations, analysis of variance with Tukey's post hoc test was used for multiple pairwise comparisons and Student’s t-test was used for between-group comparisons.

## Results

### Multilevel calibration and validation of model simulations

After the full multiscale computational model (Figure 1) was compiled, we first calibrated the dynamic post-HLI model behaviors at intracellular, cellular, and tissue levels simultaneously against a curated set of quantitative time-course experimental measurements from literature. We then validated the model, both quantitatively and qualitatively, against a number of in vitro and in vivo data regarding the multilevel biological response (eg, protein secretion, cell dynamics, perfusion recovery) in HLI experimental settings. The results of this model calibration and validation procedure are presented in the following sections.

#### Macrophage signal transduction

**Figure 2:**
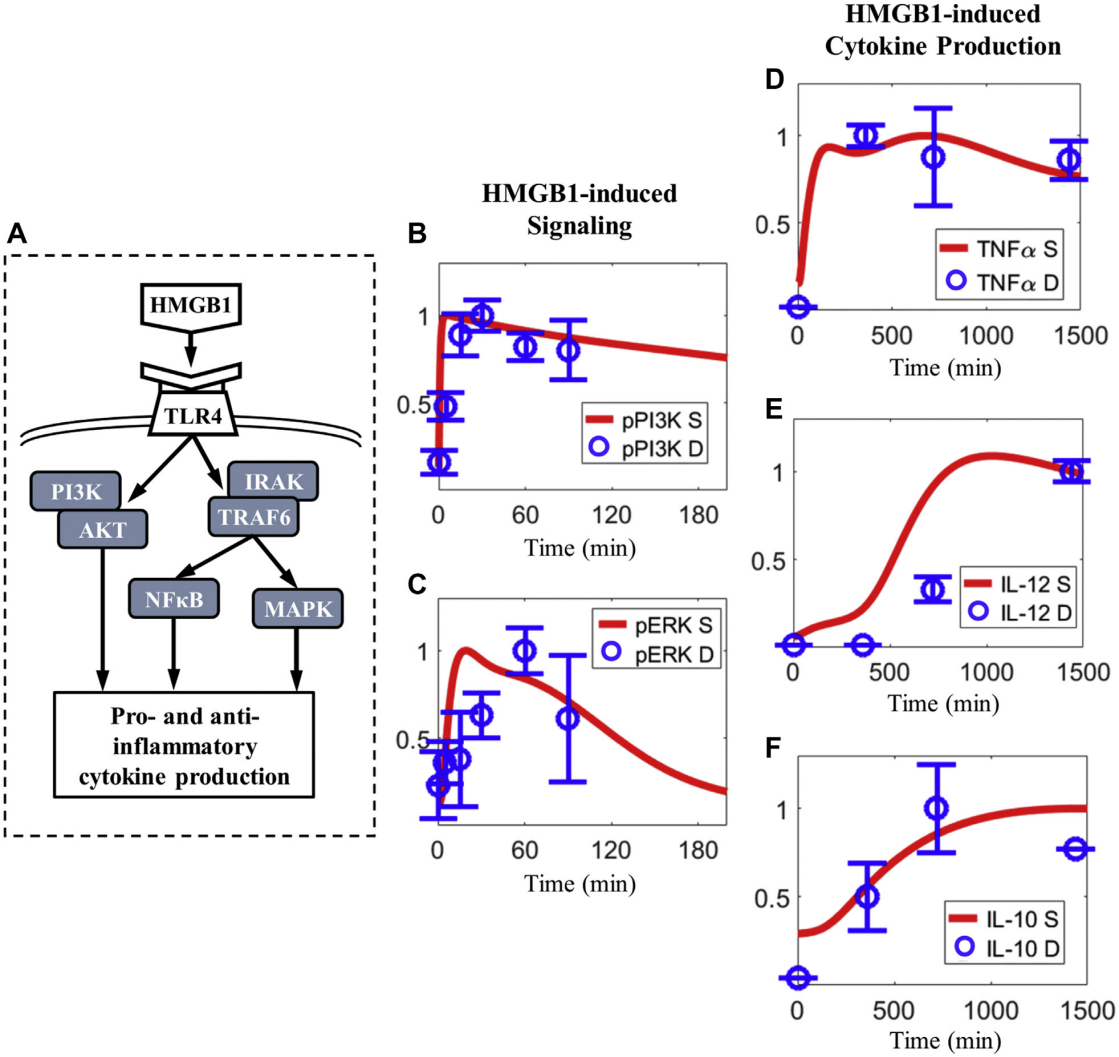
HMGB1/TLR4 Signaling Axis on Macrophages **(A)** Overview of the newly added HMGB1/TLR4 axis (full details are given in Supplemental Table 2) in the 8-pathway macrophage signaling model (see Figure 1B). **(B to C)** Relative time-course activation of phosphoinositide 3-kinase (PI3K) and extracellular receptor kinase (ERK) in response to HMGB1 stimulation in macrophages: model simulation results and corresponding experimental data (49). **(D-E)** Relative time-course production of macrophage phenotype markers, TNF-α, IL-12, and IL-10, in response to HMGB1 stimulation: model simulation results and corresponding experimental data (50). **(B to F)** All expression profiles are for protein levels and are normalized to their respective maximum values (y-axes are relative expression levels). D = experimental data; MAPK = mitogen-activated protein kinase; NF-κB = nuclear factor κB; p = phosphorylated; S = simulation; TRAF6 = tumor necrosis factor receptor–associated factor 6; other abbreviations as in Figure 1.

To accurately describe macrophage signaling in HLI, we fully incorporated the macrophage model by Zhao et al (29), which is an extensively calibrated and validated computational model that simulates 7 important signaling pathways on macrophages, and we then further added the HMGB1/TLR4 axis (the final 8-pathway model is summarized in Figure 1B). To capture the intracellular dynamics of macrophage signaling and cytokine production induced by HMGB1, we assumed that HMGB1, as a ligand, primarily interacts with TLR4 to activate downstream signaling through 2 parallel paths (Figure 2A) (51). The first path is through the phosphoinositide 3-kinase/AKT cascade, and the other path is through common TLR4 signaling adapters IL-1 receptor–associated kinases and TNF receptor–associated factor 6, which subsequently drive the activation of downstream effectors such as nuclear factor kappa B and mitogen-activated protein kinases (49,52). Thus, for the new HMGB1/TLR4 axis in the 8-pathway cell-level macrophage component, we calibrated the time-course activation dynamics of phosphoinositide 3-kinase, three mitogen-activated protein kinases (extracellular receptor kinase, p38, and c-Jun N-terminal kinase), and nuclear factor kappa B (represented by degradation of its inhibitor, IκB) in response to HMGB1 stimulation (Figures 2B and 2C, Supplemental Figure 1). Here, the addition of the HMGB1/TLR4 axis did not introduce new transcriptional regulators, nor did it alter any parameters that control the production of macrophage phenotype markers (compared with the original macrophage model). Thus, as a validation step, we simulated the profiles of 3 downstream phenotype markers (TNFα, IL-10, and IL-12) produced by macrophages under HMGB1 stimulation, and their model-predicted temporal dynamics agree well with quantitated literature data (Figures 2D to 2F) (50).

#### Cell- and tissue-level dynamics under HLI

**Figure 3:**
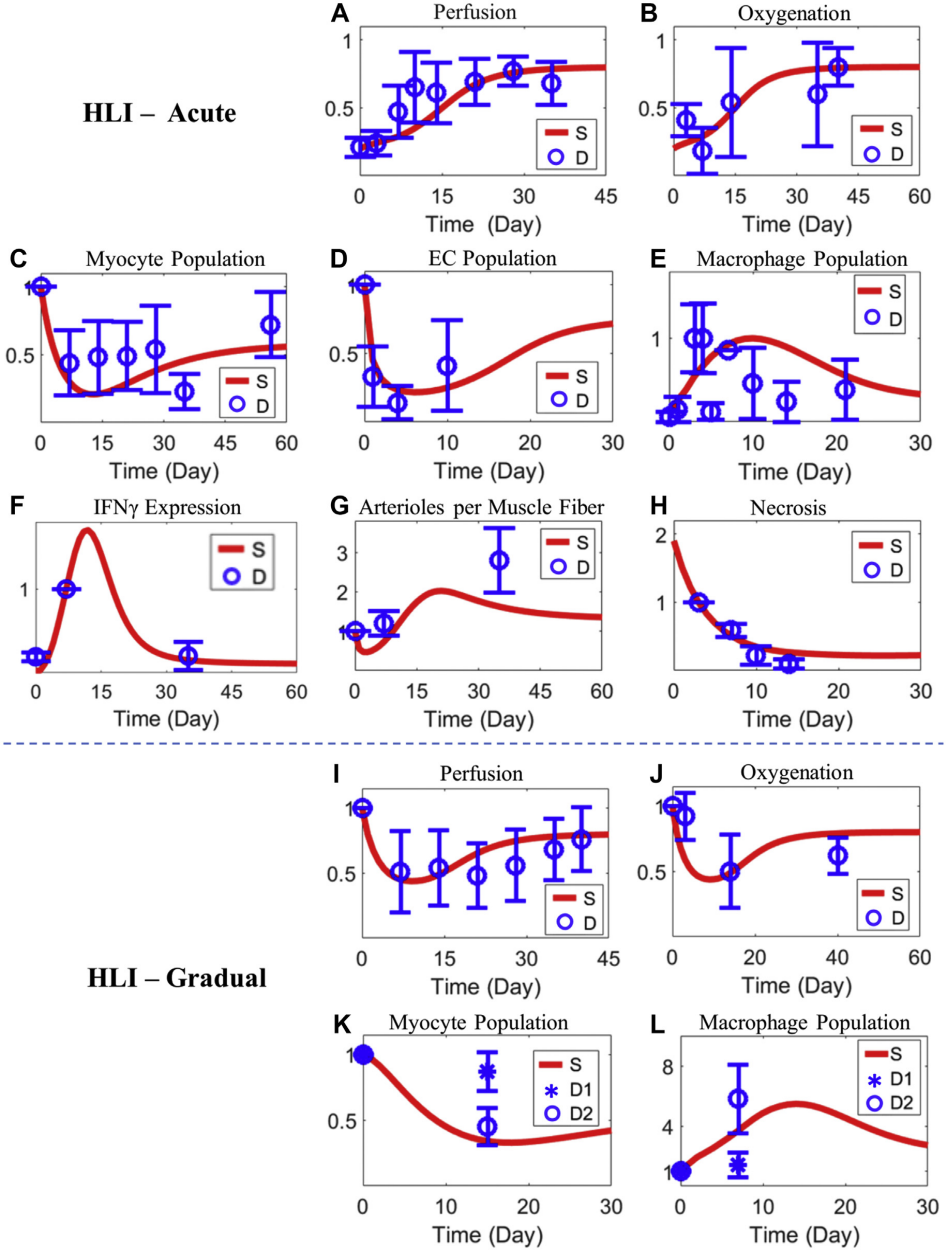
Model Calibration and Validation of Cell- and Tissue-Level Dynamics Under HLI Model simulations (“S”) and corresponding experimental data (“D”) with references are shown together. **(A)** After acute HLI, tissue perfusion gradually restores. **(B)** A similar trend has also been observed for tissue oxygenation (28,53). **(C)** Time-course dynamics of the skeletal muscle fiber size (represented by the population size of skeletal myocytes) after acute HLI (28,54,55). **(D and E)** Time-course dynamics of the population sizes of ECs and macrophages after acute HLI (20,55,56). Time-course profiles of tissue IFN-γ expression **(F)** and arterioles number per muscle fiber (28) **(G)**, and tissue necrosis after acute HLI (57) **(H)**. **(I and J)** In the settings of gradual HLI, tissue perfusion would experience a more progressive reduction in the beginning, followed by gradual recovery; a similar trend has been observed for tissue oxygenation (53,58,59). Time-course dynamics of the skeletal muscle fiber size (represented by the population size of skeletal myocytes) **(K)** and population size of macrophages **(L)** after induction of gradual HLI (58). D1 and D2 represent data collected from C57BL/6 and BALB/c mice, respectively. All values in the figure are normalized, and y-axes are relative expression levels (**A to D, G, and I to L**, normalized to the pre-HLI levels; **E**, normalized to the maximum level; **F**, normalized to the day 7 level; and **H**, normalized to the day 3 level). A complete list of references used in **A** is available in Supplemental Protocol 1. Abbreviations as in Figure 1.

We moved beyond macrophage intracellular signaling and then quantitatively calibrated the behaviors of the whole multiscale model against several cell- and tissue-level experimental observations under HLI (Figures 3A to 3E). In the commonly used acute HLI mouse model (eg, direct femoral artery ligation and transection), spontaneous recovery of tissue perfusion after the instantaneous initial drop (due to surgery) can occur over a course of several weeks; however, in later weeks, perfusion recovery usually stabilizes at a final level below the pre-ischemia level, as suggested by laser Doppler perfusion imaging (Figure 3A) (28,32,60,61). A similar trend has also been observed in the regulation of post-HLI tissue oxygenation (Figure 3B) (28,53, 54, 55, 56, 57, 58, 59). In terms of cellular dynamics, skeletal myocytes and ECs would undergo significant cell death (eg, necrosis) after the onset of acute HLI (11), and later both cells would regenerate and repopulate along with the recovery of tissue perfusion (Figures 3C and 3D). On the other hand, macrophages are initially present at relatively low levels in the muscle but are significantly recruited upon HLI-induced tissue damage and inflammation, and their population size would subsequently decrease (eg, via local death and emigration) as inflammation resolves and tissue regenerates (Figure 3E) (62,63).

After the calibration procedure, we then compared model predictions of 3 physiological outputs with corresponding experimental data obtained in HLI mice (Figures 3F to 3H) as a semi-quantitative validation step because the experimental observables are implicitly described by the model. Our model simulations suggested that tissue IFN-γ expression (Figure 3F), as an indicator of the inflammatory response, would rise sharply in the early phase of recovery and then return to baseline in the late phase (eg, at 5 weeks’ post-HLI); for tissue revascularization, the level of arterioles per muscle fiber (evaluated in our model as the ratio of population size of ECs to myocytes) is generally higher after HLI compared with pre-HLI (Figure 3G). The rate of muscle necrosis is also significantly lower in the later days compared with that in the early days after HLI (Figure 3H). Overall, these uncalibrated model predictions of cell- and tissue-level features in acute HLI have achieved good quantitative and qualitative agreements with experimentally observed trends (28,59).

We further tested the model, which was originally calibrated using only data from acute HLI settings, in the gradual HLI setting (eg, placement of ameroid constrictors) by assuming a time-dependent decrease in tissue perfusion supply as the new simulation input, based on the time-course constrictor features reported in an in vivo mouse study (Supplemental Figure 2) (30,64). All other parameters and reaction processes were kept the same as in the acute HLI simulation setting. With this new input, it was observed that the model simulations of tissue perfusion and oxygenation can still correctly predict the quantitative and qualitative trends of the corresponding in vivo experimental data shown in gradual HLI studies (Figures 3I and 3J) (53,58,59). The predicted fold changes of skeletal myocytes and macrophages after induction of gradual HLI also qualitatively fall within ranges reported in the literature (Figures 3K and 3L), despite variations in the reported data due to differences in mouse genetics (58). In summary, this prediction–validation cycle using uncalibrated gradual HLI data further strengthened the model’s underlying mechanistic formulation and established its predictive capacity in new experimental scenarios of HLI. This cycle can also be implemented iteratively to further constrain and refine the model when more experimental data become available.

#### Physiological features and impact of targeted interventions under HLI

**Figure 4:**
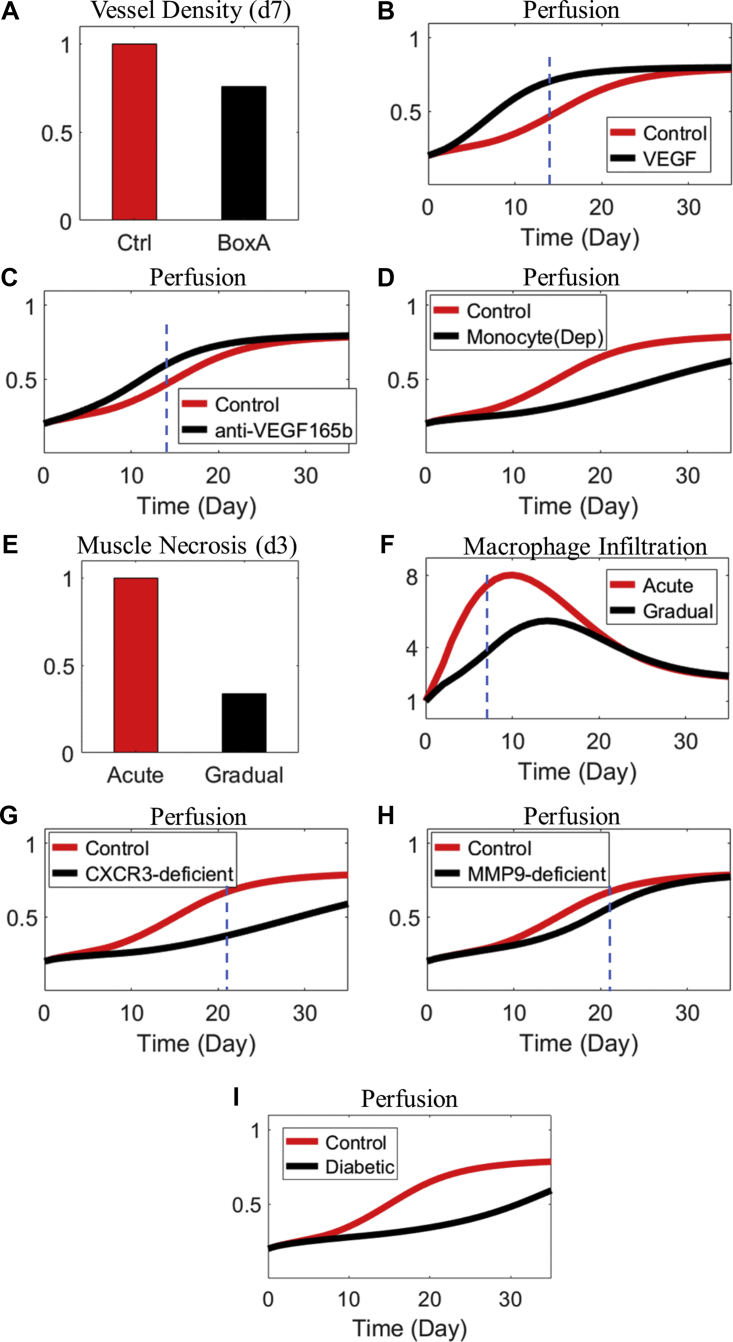
Qualitative Model Validation Against In Vivo Perturbation Data Under HLI All panels here describe simulation results, validated by findings reported in reference literature studies (as listed). **(A)** Decreased tissue vessel density at day 7 after HMGB1 inhibition using BoxA compared with control/untreated condition (“Ctrl”), as reported elsewhere (12). **(B)** Differential changes in perfusion recovery after VEGF overexpression (eg, VEGF_165_a), as reported elsewhere (44). **(C)** Inhibition of the VEGF isoform (VEGF_165_b) by antibodies, as reported elsewhere (7). **(D)** Depletion of monocytes/macrophages, as reported elsewhere (65). **(E and F)** Decreased muscle necrosis and macrophage infiltration in gradual HLI compared with acute HLI, as reported elsewhere (59). **(G and H)** Silencing of CXCR3 or MMP9 impairs overall tissue perfusion in acute HLI, as reported elsewhere (66,67). **(I)** Perfusion recovery is down-regulated in diabetic HLI (acute) compared with HLI (acute) alone, as reported elsewhere (33,34,68). All values here are normalized simulations, and y-axes are relative levels (**B to D** and **F to I**, normalized to the pre-HLI levels; **A**, normalized to the simulated day 7 level in the control condition; and **E**, normalized to the simulated day 3 level in the acute HLI condition). **Vertical dashed blue lines** in **B and C and F to H** suggest that the corresponding experimental results, as reported in the aforementioned studies, are statistically significant at the indicated time points (day 7 in **F**, day 14 in **B and C**, and day 21 in **G and H**). Abbreviations as in Figure 1.

Another important component in our multilevel model calibration and validation is to ensure that our model can correctly evaluate the impact of various targeted interventions and perturbations under HLI. For that, we compared model simulations in a number of targeted perturbation scenarios under HLI versus the corresponding experimental data in the literature, with a focus on their qualitative agreements (Figure 4) given that certain simplifying assumptions were made by our model to represent those perturbations (additional details are provided in Supplemental Protocol 1).

In acute HLI settings, our model simulations can qualitatively confirm the experimental findings regarding the impact of several perturbation strategies on post-HLI tissue vascularization and perfusion, including HMGB1 inhibition (Figure 4A) (12), VEGF_165_a overexpression (Figure 4B) (44,69), VEGF_165_b inhibition (Figure 4C) (7,70), and monocyte/macrophage depletion (Figure 4D) (65). Model simulations also reproduced the observation that activities of skeletal muscle necrosis and macrophage recruitment are both lower in the gradual HLI setting compared with the acute HLI setting (Figures 4E and 4F) (59). In addition, we qualitatively simulated the impact of 2 gene knockouts (CXCR3 and MMP9) with high relevance for the recovery of HLI, and the simulations suggested that both gene knockouts would impair perfusion recovery, which aligns with conclusions reported in the literature (Figures 4G and 4H) (66,67,71).

We also simulated the model under diabetes conditions by incorporating a representative feature of how diabetes influences the endothelium (72,73); the resulting model simulations (Figure 4I) indicated a marked impairment of post-HLI perfusion recovery in diabetic HLI conditions compared with normal HLI conditions, which agrees with the findings from a number of HLI (acute) studies (33,34,68). Again, this batch of qualitative model validation against in vivo perturbation data further expands our model’s multilevel simulation capacity.

### Dynamic macrophage phenotype spectrum and response during HLI

**Figure 5:**
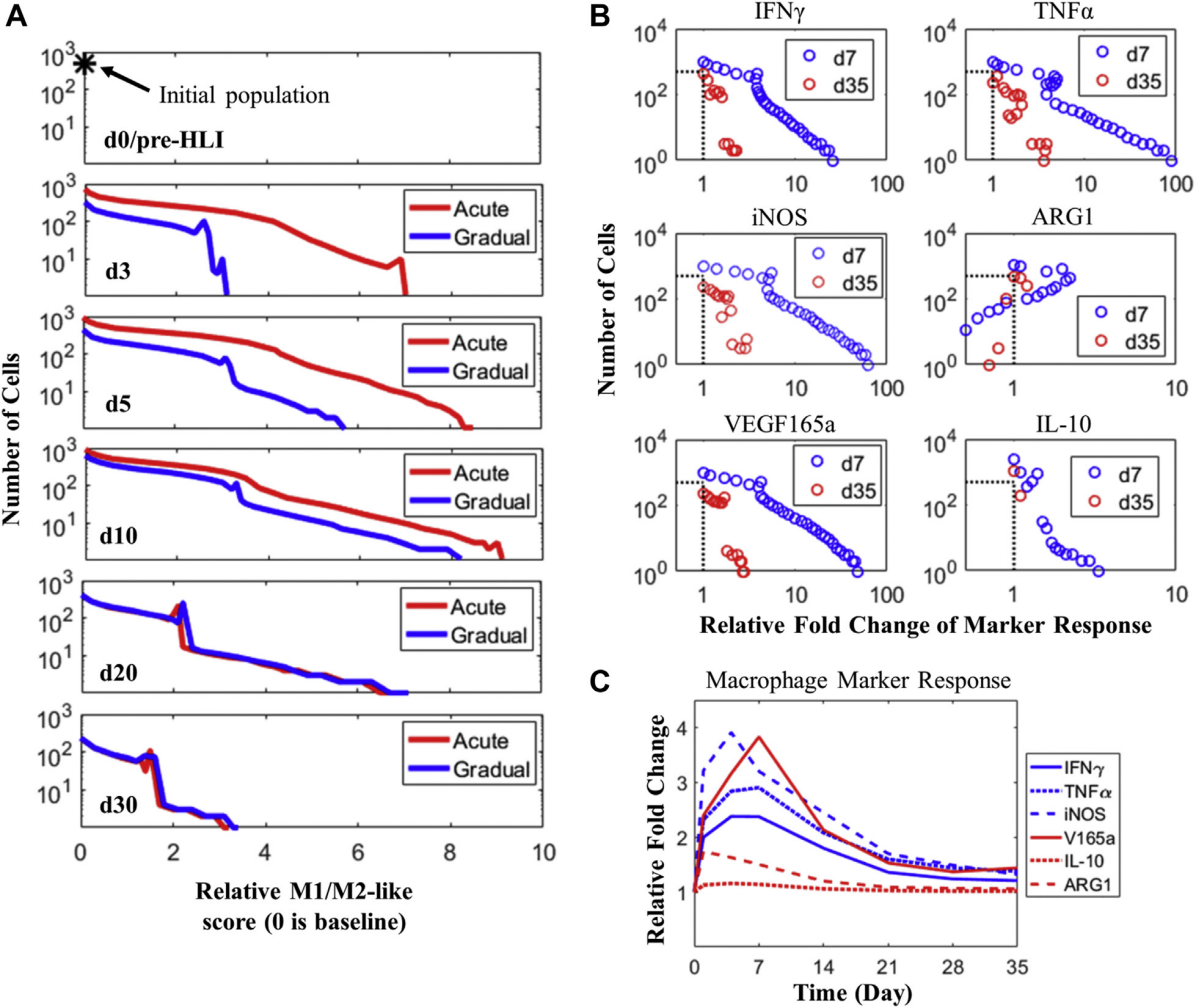
Dynamic Macrophage Phenotypes and Marker Response During HLI **(A)** Distribution of macrophage phenotype spectrums based on relative M1/M2-like scores (log_10_ transformed) at different time points during acute and gradual HLI. Raw data of all discrete macrophage populations are available in Supplemental Figure 3. **(B)** Full description of the relative regulation of six M1 and M2 markers in all macrophage populations in tissue at day 7 and day 35 after induction of acute HLI. In each panel, the intersection of the horizontal and vertical **dashed lines** indicates the size and relative marker expression of the initial macrophage population (eg, under pre-HLI/control condition). In **A and B**, the y-axes are the number of macrophages in log scale. **(C)** Temporal, cell-averaged (across all macrophage populations) expression profiles of six M1 **(blue)** and M2 **(red)** markers after induction of acute HLI. All values are normalized to the respective day 0/pre-HLI levels. ARG1 = arginase-1; d = day; iNOS = inducible nitric oxide synthase; other abbreviations as in Figure 1.

The unique multiscale formulation of the model has enabled us to probe directly and mechanistically into the dynamic phenotypes of macrophages during perfusion recovery after induction of HLI. As shown in Figure 5A, these simulations suggest that a significant overall increase occurs in the pro-inflammatory features (evaluated in terms of their relative M1/M2-like scores [the Methods section provides more details about this integrative non-dichotomous metric]) in the macrophages infiltrating the ischemic tissue during the early-phase response (eg, at day 3/5). Subsequently, in the late phase (eg, at day 20/30), the total macrophage population contracts (shown also in Figure 3E) along with a significant decrease in the overall pro-inflammatory features. Moreover, during the early phase, the degree of pro-inflammatory response as exhibited by the macrophage phenotypes is clearly much stronger in the acute HLI scenario than in the gradual HLI scenario (Figure 5A, panels 2 and 3), and this outcome is likely due to the much more rapid and drastic occurrence of tissue ischemia that directly resulted from the acute HLI surgical procedure. However, in later time points, this difference gradually diminishes; in the late phase, the macrophage phenotypes in acute versus gradual HLI converge to a rather similar profile (Figure 5A, bottom 2 panels), as suggested by our model.

Our model simulations (Figure 5A) revealed a highly continuous and spectrum-like distribution (instead of dichotomous M1 or M2 extremes) of all macrophage phenotypes in tissue based on their overall M1/M2-like scores throughout the post-HLI recovery response. We then went from the high-level M1/M2-like scores to the individual M1 and M2 markers and used the model to derive full expression profiles of these individual markers in all the macrophage populations in tissue during HLI. Based on the expression profiles of 6 representative M1 and M2 markers selected, our simulations (Figure 5B) exhibited very different degrees of regulation of both M1 and M2 markers (mostly up-regulation, except for ARG1) in early (day 7, with larger expression variations) and late (day 35, with smaller expression variations) time points.

Temporal simulations of the cell-averaged marker expression response also exhibited mixed patterns for the 6 macrophage markers (Figure 5C): for example, the three M1-type markers (IFN-γ, TNF-α, and inducible nitric oxide synthase) are time dependently up-regulated with differential intensities, whereas the three M2-type markers (VEGF_165_a, ARG1, and IL-10) do not display qualitatively consistent up- or down-regulation, which means the simple M1/M2 categorization based on a single marker is no longer valid in this case. Together, these model results, from the standpoint of both high-level M1/M2–like scores and individual markers, argue that a mechanistic consideration of a panel of relevant macrophage markers (instead of a single marker-based M1/M2 categorization), as well as the temporal and spectrum-like aspects in macrophage signaling and polarization (74), are crucial elements for the accurate understanding of the in vivo macrophage physiology and phenotypic functions in HLI.

### Model sensitivity analyses and in silico evaluation of potential strategies to improve perfusion recovery in a VMP

**Figure 6:**
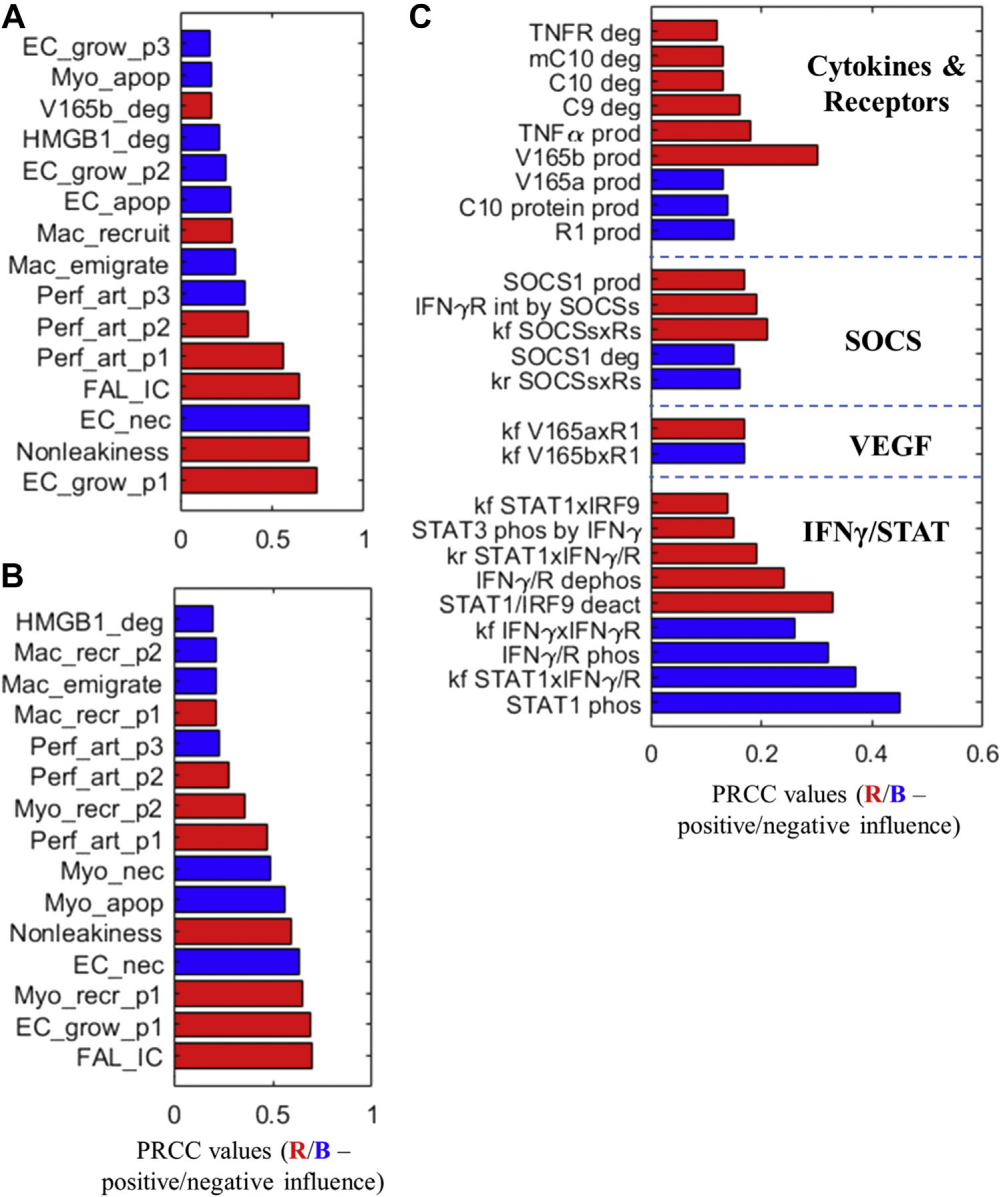
Multilevel Model Sensitivity Analyses Under HLI Conditions Sensitivity indices (**red** = positive; **blue** = negative) of the most influential (top 15) model parameters that control tissue-level perfusion (in terms of the 35-day time-course integral of perfusion) **(A)** and muscle regeneration (in terms of the 35-day time-course integral of the myocyte population size) **(B)** in acute HLI. **(C)** Sensitivity indices (**red** = positive, **blue** = negative) of the most influential (top 25) model parameters that control an antirecovery cell-level macrophage response (in terms of its 24-hour time-course integral) under hypoxia plus low-dose HMGB1. More technical details of the sensitivity analyses are available in Supplemental Protocol 1. The parameters listed in **A(labels from bottom to top**: k8, nonleakiness, k13, perfusion_IC, k9, kb9, kc9, k14, k10, k3, kb8, k15, k7, kb2, and ke8) and **B (labels from bottom to top**: perfusion_IC, k8, k1, k13, nonleakiness, k2, k12, k9, kd1, kb9, kc9, k10, k14, ka10, and k15) correspond to parameters in Supplemental Table 1. Details of the parameters listed in **C** can be found in Supplemental Table 2. PRCC = partial rank correlation coefficient; SOCS = suppressor of cytokine signaling; STAT = signal transducer and activator of transcription; other abbreviations as in Figure 1.

After our model went through multilevel calibration and validation, we then performed a series of global model sensitivity analyses at the tissue and cell levels using the partial rank correlation coefficient algorithm (additional details are provided in Supplemental Protocol 1) (48) to search for model parameters and processes that can most significantly influence perfusion recovery in HLI (acute HLI was chosen here as the representative condition for sensitivity analyses). The tissue-level analysis results in Figure 6A suggest that the parameters that control EC and macrophage growth and death are among the most influential ones that regulate tissue perfusion recovery, and very intuitively the parameters that control myocyte growth and death ranked very high in terms of their influence on time-course muscle regeneration (Figure 6B). Furthermore, to evaluate how macrophage-based perturbations can alter perfusion recovery, the results from the tissue-level analyses were summarized into one outcome measure to be optimized; the goal is to promote macrophage infiltration while decreasing its pro-inflammatory potential. We then performed sensitivity analysis at the macrophage single-cell level to determine the macrophage-specific parameters that can most significantly influence this outcome. The cell-level analysis results (Figure 6C) revealed that a number of parameters relating to macrophage intracellular signaling and polarization can potentially drive this outcome, and these parameters were categorized into 4 functional modules.

**Figure 7:**
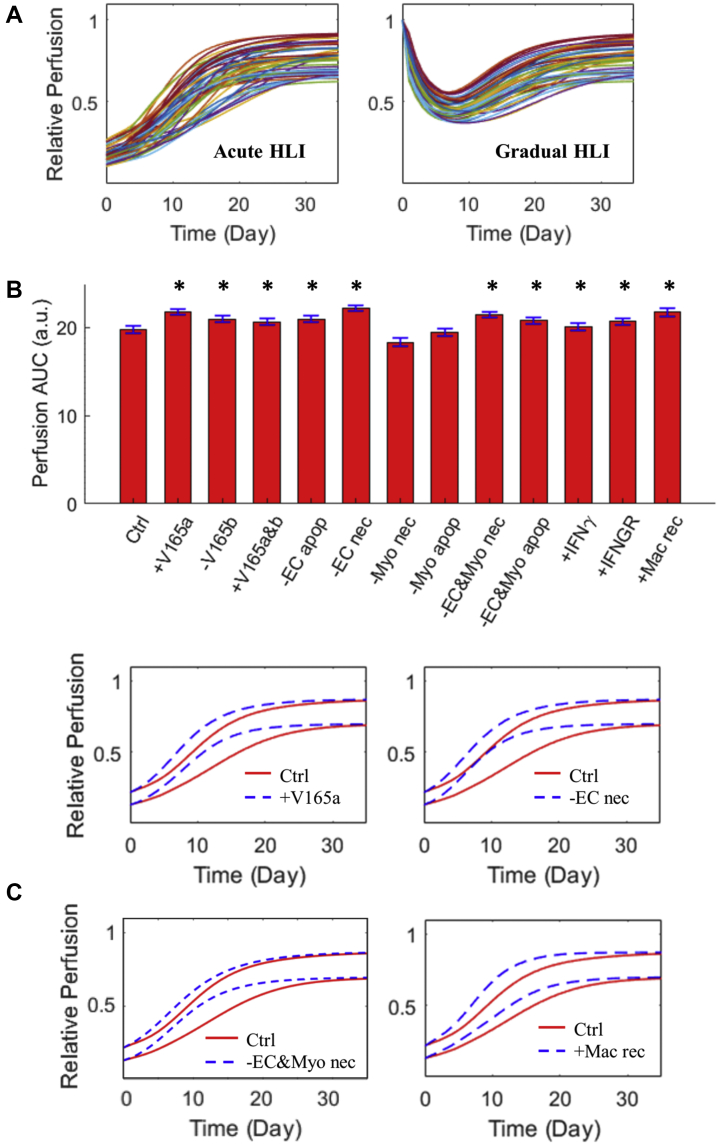
Simulated Impact of Targeted Interventions on Tissue Perfusion Under Acute HLI in a Physiological VMP **(A)** Physiological perfusion recovery responses of each virtual mice in the model-based, experimental data-driven virtual mouse population (VMP) under acute and gradual HLI conditions. **(B)** Time-course integrals of tissue perfusion in the VMP under control (Ctrl) and a number of different targeted interventions under acute HLI. For labels in **B**, “+” indicates promotion, “-“ indicates inhibition. Details regarding model implementation of these interventions are available in Supplemental Protocol 1. Integral values (in arbitrary units [a.u.]) computed from the 50 virtual mice are displayed as mean ± SEM (n = 50). ∗*P* < 0.05 compared with Ctrl with a greater mean value; 2-tailed paired Student’s t-test was used. **(C)** Time-course trajectories of mean perfusion level of the VMP (n = 50, displayed as upper/lower bounds that reflect population mean ± 1 SD) under acute HLI and with 3 interventions (labels are the same as in **B**). The corresponding full ranges of the perfusion responses in the VMP as well as responses in smaller population samples are shown in Supplemental Figure 4. apop = apoptotic death; nec = necrotic death; Myo = myocyte; Mac = macrophage; rec = recruitment; V165a/b = VEGF_165_a/b; other abbreviations as in Figure 1.

Next, we wanted to simulate and analyze how targeted model perturbations, based on results of the sensitivity analyses, will regulate perfusion recovery and muscle regeneration in acute HLI animal models at the population level. Thus, we first used our model to generate a VMP consisting of 50 virtual mice (Figure 7A) whose physiological post-HLI recovery responses (in terms of time-course tissue perfusion) are in quantitative and qualitative accordance with curated mouse data from the literature (additional details are provided in Supplemental Protocol 1 regarding generation of VMP). Then, we simulated a number of targeted interventions in this VMP and assessed the impact of these strategies on the overall perfusion recovery. As summarized in Figure 7B (and Figure 7C and Supplemental Figure 4, which contain time-course trajectories of the population mean perfusion levels), the simulations suggested that interventions such as increasing the VEGF_165_a to VEGF_165_b ratio (Figure 7C, top left), inhibiting EC death alone (Figure 7C, top right), inhibiting both EC and myocyte death (Figure 7C, bottom left), and promoting macrophage recruitment (Figure 7C, bottom right) can all enhance tissue perfusion under acute HLI in the VMP; inhibiting myocyte death alone, however, has no positive effects on perfusion. Interestingly, controlled promotion of IFN-γ signaling on macrophages (eg, by low-dose, short-term administration of IFN-γ) can also increase tissue perfusion according to our simulations, albeit the effect is less pronounced compared with other strategies (Figure 7B). To our knowledge, the idea of promoting perfusion through controlled IFN-γ pathway activation has not yet been tested in HLI settings; however, a relevant study by Spiller et al (75) found that low-dose short-term IFN-γ release by engineered scaffolds can enhance vascularization in a subcutaneous implantation model in mice and that macrophages may be a key node to achieve this effect. In addition, all strategies were suggested to enhance overall myocyte survival and regeneration to different degrees (Supplemental Figure 5).

### Validation of model-predicted therapeutic intervention in HLI mice

**Figure 8:**
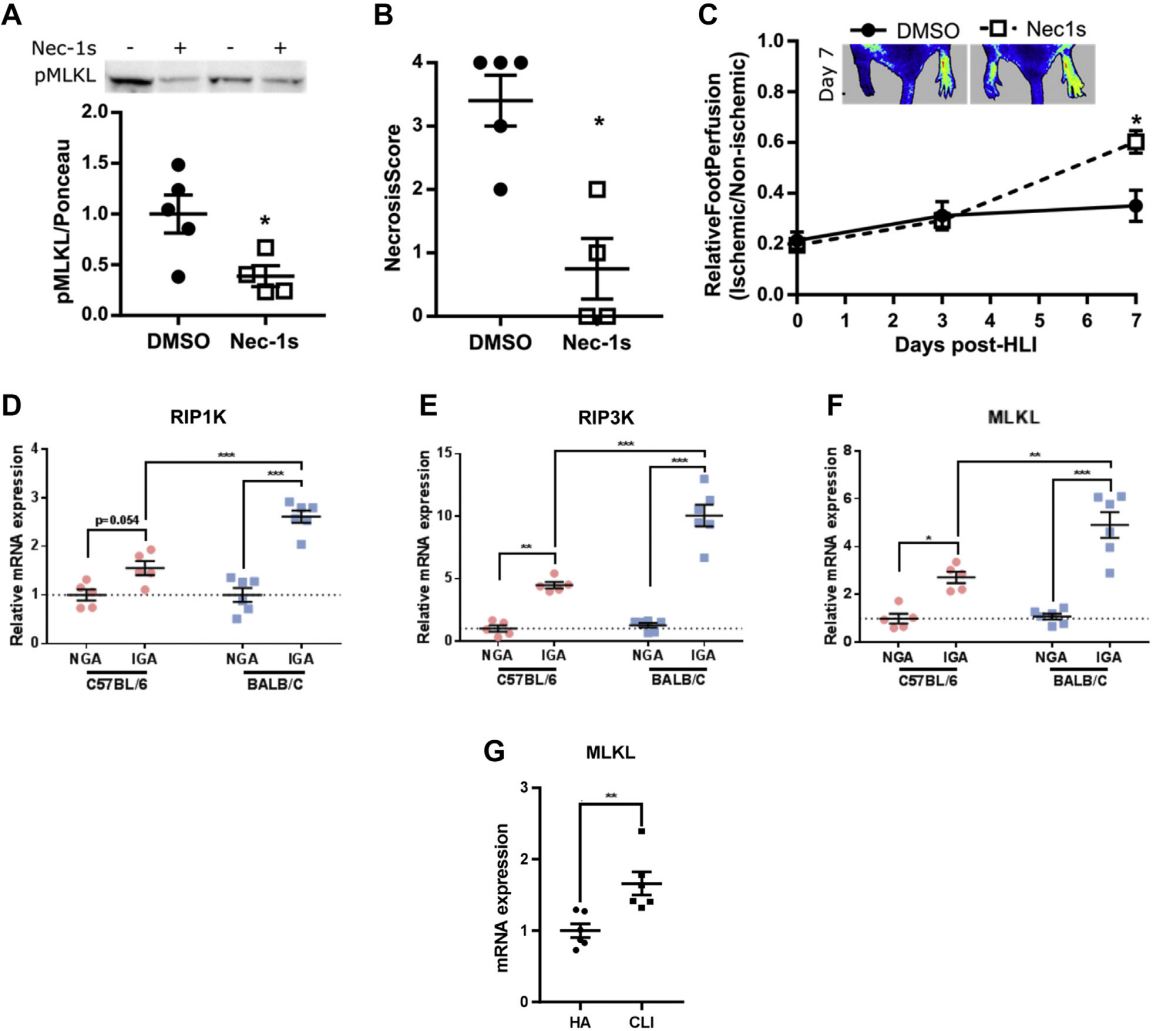
Inhibition of Programmed Necrosis by Nec-1s Improves Limb Survival and Early Blood Flow Recovery After HLI **(A)** Protein expression by western blot of phosphorylated mixed lineage kinase domain-like protein (pMLKL) in ischemic gastrocnemius muscles (IGA) of BALB/c mice treated with either 1.6 mg/kg/d of Nec-1s (n = 4) or vehicle control (0.05% dimethyl sulfoxide [DMSO], n = 5) intraperitoneally for 7 days after HLI surgery. **(B)** Cumulative gross necrosis score of ischemic limb of BALB/c mice after HLI. **(C)** Blood flow recovery via laser Doppler perfusion imaging up to 7 days’ post-HLI. Relative messenger RNA (mRNA) expression of RIP1K **(D)**, RIP3K **(E)**, and MLKL **(F)** on day 3 after HLI in both the nonischemic gastrocnemius muscle (NGA) and IGA in 2 different mouse strains, C57BL/6 (n = 5) and BALB/c (n = 6). Importantly, the BALB/c strain is well known to exhibit more severe necrosis and poorer outcomes than C57BL/6. **(G)** Relative mRNA expression of MLKL in limb biopsy samples of patients with critical limb ischemia (CLI) (n = 6) compared with healthy adults (HA) (n = 6). ∗*P* < 0.05, versus DMSO control by 2-tailed Student’s t-test **(A and B)**; by 2-way analysis of variance followed by Tukey’s post hoc test for multiple comparisons **(C and F)**. ∗∗*P* < 0.01, by 2-way analysis of variance followed by Tukey’s post hoc test for multiple comparisons **(E and F)**; by 2-tailed Student’s t-test **(G)**. ∗∗∗*P* < 0.001, by 2-way analysis of variance followed by Tukey’s post hoc test for multiple comparisons **(D to F)**.

From the model sensitivity analyses and VMP simulations presented earlier (in Figures 6 and 7 and Supplemental Figure 5), we see that inhibition of cell (EC and myocyte) necrotic death is predicted to be among the top-ranked therapeutic interventions that can potentially promote perfusion recovery and improve myocyte survival and regeneration after acute HLI; particularly, this proposed strategy can be pharmacologically realizable in vivo, and it had not been previously evaluated in animal models. Driven by this finding, we tested the therapeutic effect of this strategy in HLI by intraperitoneally injecting Nec-1s, a potent necroptosis (a regulated form of necrosis) inhibitor, into BALB/c mice subjected to HLI. Compared with control mice (treated with dimethyl sulfoxide), Nec-1s treatment in vivo reduced the activation of MLKL, the terminal executioner of the necroptotic cell death pathway, in the ischemic limb tissue (Figure 8A). Nec-1s–treated mice also exhibited significantly improved perfusion recovery as well as reduced skeletal muscle necrosis after HLI (Figures 8B and 8C), which validates the aforementioned model prediction.

In addition, we found that the gene expression of 3 key effector molecules responsible for necroptosis (RIP1K, RIP3K, and MLKL) are all significantly elevated at day 3 post-HLI in the ischemic limb compared with control (nonischemic) limbs in BALB/c mice (Figures 8D to 8F). This evidence indicates the presence of an early-phase activation of ischemia-induced necroptotic/necrotic cell death programs within the tissue after HLI (also supported by in vitro data under the condition of hypoxia serum starvation, as shown in Supplemental Figure 6). Furthermore, in human PAD settings, the expression of MLKL is also elevated in limb muscle samples from patients with critical limb ischemia compared with healthy controls (Figure 8G). Taken together, the mechanistic model-based computational analyses combined with experimental validation and data presented here, for the first time, highlight the pathophysiological importance and potential translational value of targeting this necrotic cell death axis in the treatment of PAD.

## Discussion

The current paper presents the first computational model that quantitatively describes the postischemic recovery response in the pathophysiology of PAD; more specifically, our model, which has been systematically calibrated against intracellular, cellular, and tissue-level experimental data, can mechanistically simulate a number of multiscale and multicellular signaling and regulatory processes that dynamically contribute to perfusion recovery in HLI, a commonly used animal model for PAD. In addition to the simulated level of perfusion recovery as the key model readout for tissue revascularization (which is a major focus in preclinical studies of PAD), our model describes several cellular and cytokine readouts (eg, number of total skeletal myocytes, tissue level of IFN-γ and TNF-α, muscle necrotic rate) that may be linked to the physiological and functional status of the recovering tissue postischemia.

In clinical studies, a very important endpoint and direct measure of functional performance in patients with PAD is the subject’s ability to exercise (eg, peak walking time, 6-minute walking distance) (76). However, given the very short duration of exercise tests (usually on the scale of minutes, for both human and mouse), cellular metabolic activities (eg, mitochondrial function) rather than tissue-level remodeling (which usually takes days and weeks) is the more likely dominating factor that determines a subject’s performance (77,78). Moreover, it has been reported that the extent of mitochondrial dysfunction in patients with PAD is surprisingly uncoupled from their calf tissue perfusion status, although both factors are correlated with a patient’s exercise performance (79). This evidence again points to the multifactorial and multiscale nature of PAD pathophysiology; from the modeling aspect, a parallel axis that describes regulation of mitochondrial bioenergetics shall be added in our future model developments to establish a formal link between perfusion, metabolism, and exercise performance in PAD to further enhance the model’s translational impact.

The model currently describes 3 major cell types and their communications in the ischemic limb muscle tissue: skeletal myocytes to represent the muscle fibers, ECs to represent the vasculature, and macrophages to represent the immune cell infiltrates. This core module (of 3 cell types) can certainly be further enriched to allow a more complete characterization of the complex biology involved in tissue revascularization and regeneration in PAD settings (eg, HLI), as recent single-cell studies have shown that the lower limb skeletal muscle tissue of human and mouse is composed of a very wide spectrum of different cell types (23,24,80, 81, 82); however, few of them (other than myocytes, ECs, and macrophages) have been thoroughly examined for their functional roles in PAD.

T cells are another class of immune cells with relatively clear regulatory functions in PAD. In HLI, both CD8^+^ and CD4^+^ T cells were found to positively influence collateral vessel development and perfusion recovery (in part through the recruitment of macrophages) (83,84). The presence and induction of regulatory T cells were also important for postischemic revascularization through pathways possibly mediated by IL-10 (85). Among the nonimmune cells in the lower limb skeletal muscle tissue, fibroblasts and fibroblast-like cells (eg, fibro-adipogenic progenitors, tenocytes) are present at very high abundance (23). After ischemic injury, some plastic fibroblast subsets reportedly have the capacity to transdifferentiate into endothelial lineage cells and enhance capillary formation and limb perfusion (86). Fibroblasts are also critically involved in extracellular matrix remodeling and fibrosis, and persistent pathologic fibrosis is detrimental in postischemic muscle repair because it leads to impaired muscle contraction (60). Vascular smooth muscle cells also play critical roles in the maintenance of vascular function, and recent studies have shown that injection of stem cell–derived smooth muscle cells can improve angiogenesis and perfusion in HLI through pathways involving cytokine and cellular communication (10,87). In short, future efforts that aim to drive this systems-level modeling of PAD forward should carefully examine and incorporate such cell type knowledge (eg, on T cells, fibroblasts, and smooth muscle cells in PAD); thus, more mechanistic aspects of the skeletal muscle tissue microenvironment can be accounted for and precisely integrated into the model-based quantitative assessment of postischemic perfusion and functional recovery.

In the field of PAD, our model is the first quantitative modeling study that mechanistically considers multilevel time-course experimental data during calibration of its dynamic model behaviors in the setting of tissue ischemia. However, several other computational models have also been previously developed to study other biological phenomena and processes of high importance for PAD pathophysiology. For example, 2 related agent-based models by Virgilio et al (88) and Martin et al (89) focused primarily on acute muscle injury (nonischemic) and regeneration; in their models, the authors included a number of immune and nonimmune cell types to mechanistically describe the multicellular kinetics and interactions during muscle damage, inflammation, resolution, and regeneration in simulated 2-dimensional fascicle cross-sections. Ji et al (90) and Mac Gabhann et al (91) used a modeling framework based on diffusion equations and ordinary differential equations to study how oxygen and VEGF are spatially transported and distributed within the skeletal muscle tissue during exercise to influence angiogenesis. At the whole-body scale, systems pharmacology models by Chu et al (92) and Clegg et al (93) revealed how various pro-angiogenic and anti-angiogenic VEGF isoforms (VEGF_165_a and VEGF_165_b) are differentially distributed within body compartments (eg, blood, calf muscle) in PAD as well as the resulting impact on downstream proliferative signaling in muscle.

Our work, therefore, as an initial data-driven modeling attempt to explore the multidimensional biological complexity in limb ischemia in PAD, can likely be further enriched and integrated (eg, through carefully designed multiscale modeling platforms) to interface with the essential features of these prior models of different classes and enable a more comprehensive coverage of mechanistic details, simulation resolution, and predictive capacity to better probe into the pathophysiology of PAD (94). Our work presented here can also serve as a starting basis for the future construction of quantitative systems pharmacology-like platform models of PAD in which researchers can add in disease-relevant cells and pathways in modularized manners and efficiently analyze potential drug candidates by using platform-generated virtual subjects (eg, patients, animals) to speed up bench-to-beside translation (95).

### Study limitations

In addition to the general model scope-related limitations as mentioned in the Discussion section, a few technical aspects are worth further consideration and exploration by future experimental and modeling efforts. First, the current model formulation assumed a uniform distribution of cells (and their states) within a simulated bulk tissue during ischemia, whereas in PAD settings, the extent of tissue damage, perfusion, and patient survival varies depending on the disease location (eg, proximal vs distal PAD) (96). One model-based approach to describe this heterogeneity and incorporate this translational aspect is to add additional compartments that correlate with different parts (eg, major muscle) of the lower limb, while each compartment contains a set of reactions that describe the relevant cell- and tissue-level regulation. This approach will allow a more accurate and muscle-specific description of blood flow distribution, impact of occlusion in different arteries, dynamics of cells and biomolecules (eg, cytokines), and postinjury revascularization and regeneration. The second technical aspect is the use of appropriate model reduction techniques to mechanistically describe time-dependent signaling events and cellular heterogeneity while maintaining a reasonable model computational cost in a deterministic simulation framework, as cellular heterogeneity (eg, polarization spectrum) is an important feature for macrophages and fibroblasts that both play significant roles in the pathophysiology of PAD. To manage this issue, appropriate surrogate models (of cell signaling) may be created (eg, by machine learning) to reduce the time-dependent signaling complexity and enable faster simulations (97). An alternative approach is to explicitly enumerate the relevant stimulus-output dose–response relationships (at assumed steady states) from the original complex mechanistic signaling models and then construct simpler models with only that dose response information. The third aspect is about the generation of model-based virtual subjects (eg, virtual mice). The current virtual subject selection criteria implemented only focused on the high-level perfusion recovery response; future modeling studies that aim to perform similar analyses can include further considerations of the mouse strain differences and additional screening criteria such as constraints of cell/cytokine dynamics, preclinical and/or clinical data–derived predefined ranges for initial parameter resampling, and matching of population-level strain-specific perfusion response characteristics.

## Conclusions

To the best of our knowledge, this study presents the first computational model that characterizes the complex PAD pathophysiology in a preclinical setting at multiple scales. Our findings demonstrate its potential for extracting dynamic cell- and tissue-level features, identifying and assessing novel therapeutic interventions, and guiding experimental designs for future animal studies.

Perspectives **COMPETENCY IN MEDICAL KNOWLEDGE:** Regulation of limb perfusion recovery is central to the management of ischemic symptoms of PAD, for which therapeutic development has been unsuccessful for the past 2 decades. The findings from this study show that the novel systems biology approach that integrates knowledge across scales and studies can create a translational in silico platform to better understand the complex multiscale and multicellular nature of perfusion recovery in PAD and uncover new potential therapeutic opportunities.

**TRANSLATIONAL OUTLOOK:** Our work opens up a new avenue to use mechanism-based systems biology modeling to advance translational research in PAD. Future studies should aim to further expand our modeling platform by incorporating additional disease-associated cells, mechanisms, and pathways as well as preclinical and clinical data. This approach would help bridge the gap between PAD experimental research in mouse and human and facilitate a more solid framework of model-informed therapeutic discovery.

## Funding Support and Author Disclosures

This work was supported by the National Institutes of Health (grants R01HL101200, Drs Popel and Annex; R01HL141325, Dr Annex) and the American Heart Association (grant 19PRE34380815, Dr Zhao). Part of this research was conducted by using computational resources at the Maryland Advanced Research Computing Center. The funders had no role in study design, data collection and analysis, decision to publish, or preparation of the manuscript. The authors have reported that they have no relationships relevant to the contents of this paper to disclose.
