# Supplementary material for: Dynamic Multiscale Regulation of Perfusion Recovery in Experimental Peripheral Arterial Disease: A Mechanistic Computational Model
Source: JACC Basic Transl Sci. 2022 Jan 5;7(1):28–50. doi: 10.1016/j.jacbts.2021.10.014 (PMC8807862; doi:10.1016/j.jacbts.2021.10.014)
Supplement: Supplemental Data [file mmc1.pdf]

Supplemental Materials

Dynamic multi-scale regulation of perfusion recovery in experimental peripheral arterial disease: a mechanistic computational model

Chen Zhao, Joshua L. Heuslein, Yu Zhang, Brian H. Annex, Aleksander S. Popel

This compiled PDF includes the following items:

Protocol S1: Additional details of model formulation, simulation and analysis…………………2

Figure S1: Additional quantitative calibration of the HMGB1/TLR4 axis in the cell-level model component……………………………………………………………………………………6

Figure S2: Estimation of ameroid constrictor size change over time *in vivo*…………………….6

Figure S3: Raw simulation data for the analysis of macrophage phenotype spectrums in hindlimb ischemia (HLI)………………………………………………………………………7

Figure S4: Time-course perfusion response in the virtual mouse population (VMP)……………8

Figure S5: Simulated impact of targeted interventions on myocyte survival and regeneration under acute HLI in the virtual mouse population (VMP)…………………………………………9

Figure S6: Necroptosis pathway expression is increased in endothelial cells exposed to hypoxia and serum starvation……………………………………………………………………….10

Table S1: Details of model reactions, initial conditions and parameter values of the tissue-level model component……………………………………………………………………………...11

Table S2: Details of model reactions, initial conditions and parameter values of the cell-level (macrophage) model component……………………………………………………………....18

Table S3: Summary of literature data used in model calibration and validation………………..21

Protocol S1: Additional details of model formulation, simulation and analysis

Tissue-level reaction mechanisms and kinetics

Most of the tissue-level reactions relating to cell growth and death (e.g. TNFα-mediated endothelial cell apoptosis) were assumed to happen in indirect Hill-type manners (reactants such as cytokines were not directly consumed). The parameters used in Hill-type reaction rates were either estimated from literature dose-response data or fitted (see Table S1). The underlying mechanistic bases behind all modeled reactions were summarized in Table S1. It should be noted that some data that we used to formulate and evaluate the model mechanisms and reactions are not from skeletal muscle settings (e.g. data describing macrophage signaling are from *in vitro* conditions).

For the calculation of perfusion deficit used in many processes (e.g. endothelial cell necrosis), we used the numerical difference between 1 and 'true perfusion', which equals 'perfusion' times 'nonleakiness' ('perfusion' and 'nonleakiness' are both explicit model species, while 'true perfusion' is just a product). Also, the notion of perfusion shown in all figures are actually the computed 'true perfusion' values normalized to the pre-HLI value (of 1). The value of tissue oxygenation was assumed to be directly proportional to 'true perfusion' by a scaling factor. In the evaluation of literature data, multiple values of the same observable derived from different experimental studies were first processed using the mathematical method described in (1) before being used in model calibration and validation.

Generation of the virtual mouse population (VMP)

To generate qualified virtual subjects in the VMP, we first sampled (using the uniform random sampling method) a predefined parameter space (see Table S1 for more details) to generate a candidate parameter set. Then we ran the model with this newly sampled parameter set and filtered out this set if it did not generate a qualified response (e.g. trajectory did not fall inside the reasonable range) under acute and gradual HLI conditions. The reasonable response range for acute HLI was computed based on the time-course perfusion data in a collection of literature

studies (2-10) (an overall mean and standard deviation were obtained, and the reasonable range is defined as mean$\pm$1.5 SD; this information was also used in Figure 3A as mean$\pm$SD in the model calibration step). Similarly, a reasonable response range for the gradual HLI condition was also computed (11-13). Then this process of sampling and rejecting was repeated until we have generated 50 qualified virtual mice.

Simulation of targeted interventions

To qualitatively simulate the impact of different targeted interventions shown in Figure 4, we made the following simplifying assumptions (some based on relevant literature knowledge) to incorporate these interventions into the model framework: 4A – administration of boxA was implemented as a 95% reduction in HMGB1 availability (14), 4B – VEGF overexpression was implemented as 10x the baseline $VEGF_{165}$a secretion rate in myocytes and endothelial cells (15,16), 4C – anti-VEGF165b was implemented as a 95% reduction in VEGF165b availability, 4D – monocyte depletion was implemented as a 90% reduction in all baseline macrophage recruitment rates, 4G – CXCR3 deficiency was implemented as the loss of CXCL9/10-mediated induction of macrophage recruitment (17), 4H – MMP9 deficiency was implemented as 0.1x the availability of $VEGF_{165}$a (18). Note that these model implementations may consider only part of the mechanisms of action of these interventions, given the limitations set by the current model scope. Similarly in 4I, the effect of diabetes was implemented semi-mechanistically as a 40% increase in EC apoptosis rate and a 10% decrease in EC proliferation rate, according to the *in vitro* findings from (19); still, this is only one of the many aspects that diabetes may influence perfusion recovery in experimental PAD settings. In addition, all simulations in Figure 4 assume no change in the default model initial conditions (at t=0) due to these interventions.

Implementation of the different targeted interventions in our model, as listed in Figure 7, were as follows: +V165a – 10x the baseline secretion rate of $VEGF_{165}$a from endothelial cells and myocytes, -V165b – 95% reduction in the availability of $VEGF_{165}$b, +V165a&b – 7x the baseline secretion rate of $VEGF_{165}$a and 4x the baseline secretion rate of $VEGF_{165}$b from endothelial cells and myocytes, -EC apop – 0.1x endothelial cell (EC) apoptosis rate, -EC nec – 0.1x EC necrotic death rate, -Myo nec – 0.1x myocyte necrotic death rate, -Myo apop – 0.1x myocyte apoptotic death rate, -EC&Myo nec – 0.1x EC and myocyte necrotic death rate, -EC&Myo apop – 0.1x EC

and myocyte apoptotic death rate, +IFNγ – addition of IFNγ for two days at 30000 molecules/cell each day, +IFNGR – 10x IFNGR production rate in all macrophages, +Mac rec – 5x the recruitment rate for all macrophages. In the simulations, the default duration of these changes is the entire simulation timespan unless noted otherwise (e.g. in the case of +IFNγ).

Calculation of model initial conditions

Here we discuss how we derive initial conditions for modeled variables. For the very first model initial conditions (day 0 values in figures), regarding the three cell types modeled, we assumed that skeletal myocytes make up 90% of all cells within a skeletal muscle tissue (e.g. hindlimb), while for the remaining mononuclear cells, approximately 30% are ECs and 5% are macrophages, based on a recent single-cell profiling study of mouse tibialis anterior (20). Using this relative ratio information, we then set initial conditions for the three cell types in a simulated tissue volume that contains 100000 cells (in our model, the absolute numbers of cells are less important as they are always normalized during computation). For tissue oxygenation, the first initial condition is set to 6% O_2 given *in vivo* ranges reported in literature (21-24). The first initial conditions for $VEGF_{165}$a & b were calculated using the corresponding secretion rates and degradation rates. Initial HMGB1 level is set to 0. Perfusion under control condition was set to 1 at day 0 (for acute HLI, this was set to 0.25 at day 0 given that many studies have captured a residual perfusion level close to that value even immediately after HLI surgery; for gradual HLI, we also set a final residual perfusion of 0.25 and the time-course perfusion decrease was estimated using the relationship shown in Fig.S2). For most simulations, we set nonleakiness to 1 (e.g. not leaky) under control condition; for acute/gradual HLI, this was set to 0.8 (e.g. leakage) starting from day 0 till the end of simulation given the observation that many HLI mice will not reach 100% effective perfusion even in the long run, compared to normal controls. For the macrophage cell-level model, its first initial conditions were available in Table S2. Under the control condition, the most important model outputs (e.g. cells, perfusion, oxygenation) were all at relative equilibrium.

For the computation of model initial conditions at each subsequent interval, all macrophage populations have their new initial conditions based on the end results from the previous interval (the newly recruited population at each interval uses one set of default initial conditions). Since

the cell-level and tissue-level components are simulated in parallel, the cytokine initial conditions in the tissue-level component were also based on the end results (from both cell- and tissue-level components) from the previous interval and then normalized to a molecules per cell basis. The initial conditions of other species (e.g. cells, perfusion, oxygenation) directly used the end results from the previous interval. Full details of how these initial conditions were computed are described in the sample MATLAB script available online in our GitHub repository.

Model sensitivity analyses

Sensitivity analyses were performed using the PRCC algorithm (and code) as described in Marino et al [(25)]. For the algorithm settings in the tissue- and cell-level analyses, we used Latin Hypercube Sampling (LHS), p=0.05 as the cutoff for statistical significance, 220 iterations for the tissue-level analysis (5000 iterations for the macrophage cell-level analysis), evaluation time integral of day 0 to day 35 for tissue-level analysis (time integral of t=0 to 24 hr for the macrophage cell-level analysis), simulation interval of 24 hours/1 day for the tissue-level analysis, and 0.5x-2x as the allowed ranges for all parameter value variations. The product of macrophage-produced $VEGF_{165}b$, TNFα and IFNγ divided by the product of $VEGF_{165}a$, CXCL9 and CXCL10, as an indicator of an anti-recovery phenotype in macrophages based on the conclusions from the tissue-level analysis, is chosen as the output of interest (e.g. to be minimized) for the macrophage cell-level sensitivity analysis.

Figure S1

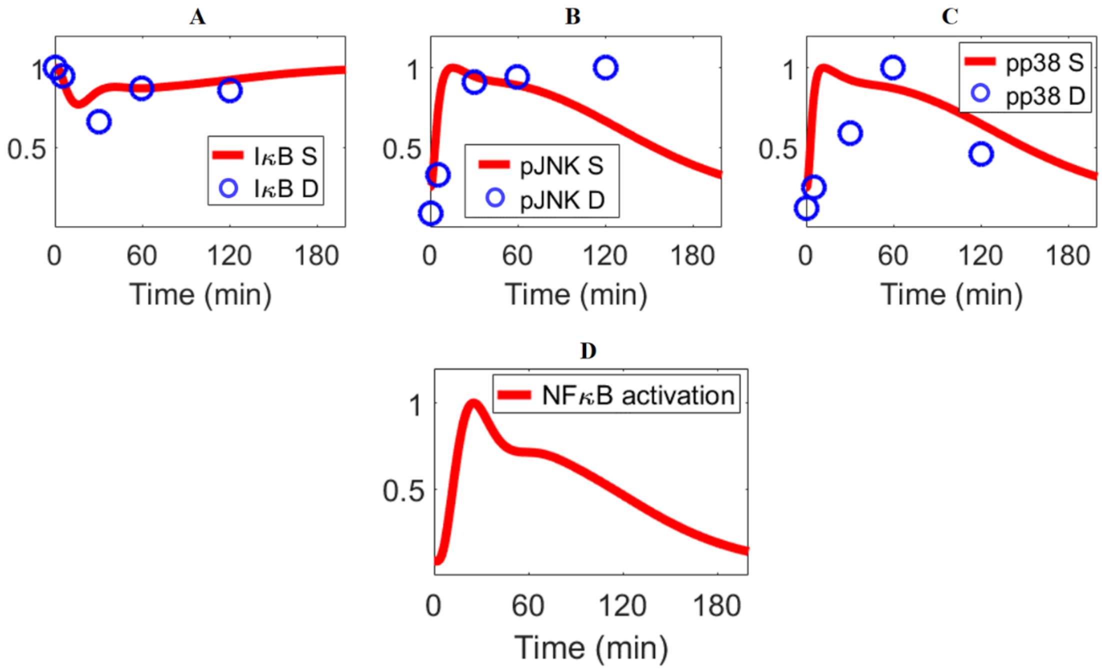

Figure S1. Additional quantitative calibration of the HMGB1/TLR4 axis in the cell-level model component. (A-C) Relative time-course activation of NFκB (represented by IκB degradation), JNK, and p38 in response to HMGB1 stimulation: model simulation results ('S') and corresponding experimental data ('D', from fibroblasts) (26). (A-D) All values are normalized and y-axes are relative expression (B-D: normalized to the respective maximum levels; A: normalized to the untreated/control level at time 0).

Figure S2

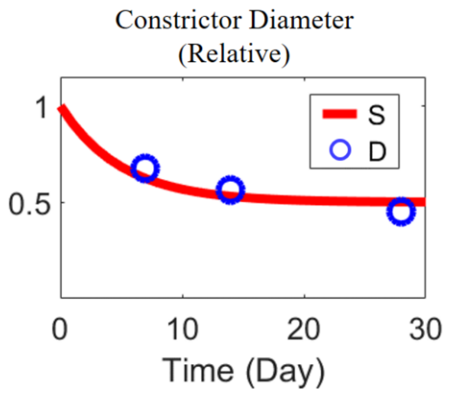

Figure S2. Estimation of ameroid constrictor size change over time *in vivo*. Time-course shrinkage of constrictor inner diameter over time: model simulation ('S') and *in vivo* experimental data from mice ('D') (27). This estimated relationship was then used to calculate the time-dependent model inputs for the gradual HLI simulations (see Protocol S1 for more details).

Figure S3

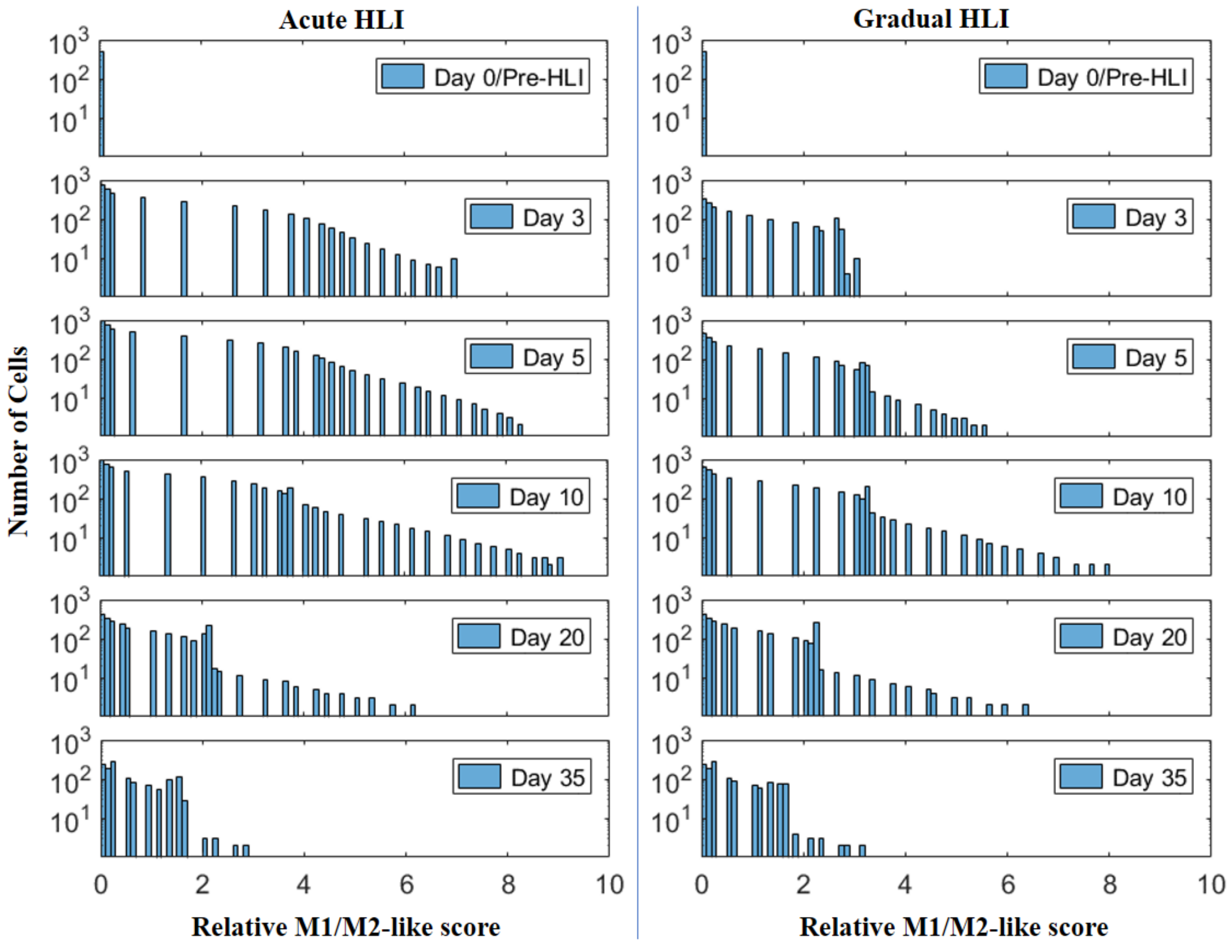

Figure S3. Raw simulation data for the analysis of macrophage phenotype spectrums in HLI. All discrete macrophage populations with their sizes and associated relative M1/M2-like scores (log10 transformed) are depicted (as histograms) at multiple time points after induction of acute (left) and gradual (right) HLI.

Figure S4

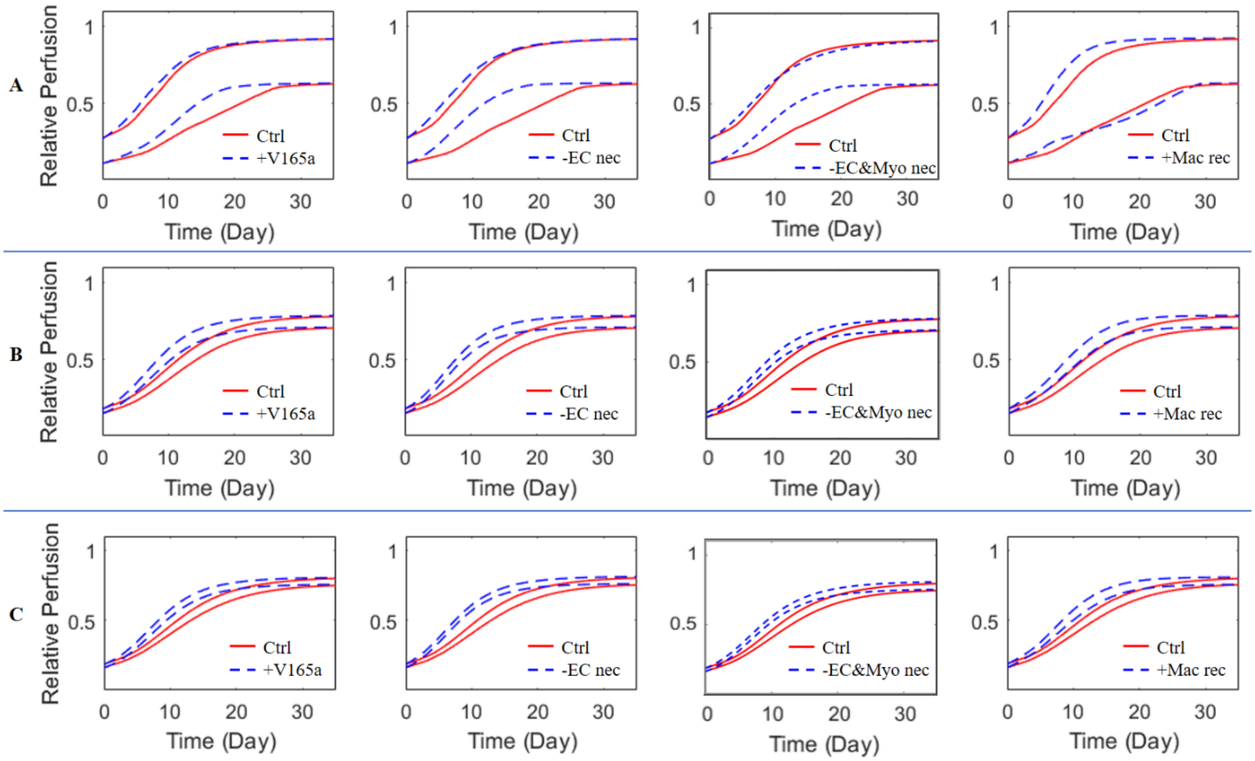

Figure S4. Time-course perfusion response in the virtual mouse population (VMP). (A) Full ranges (displayed as upper and lower bounds) of the perfusion response in all 50 virtual mice under acute HLI ('Ctrl') and HLI plus interventions. (B) Perfusion response in a representative sample of 10 virtual mice under acute HLI and HLI plus interventions (displayed as upper/lower bounds that correspond to the sample mean plus/minus one SEM). (C) Distribution (based on 100 random samples of n=10) of the sample perfusion response under acute HLI and HLI plus interventions (displayed as upper/lower bounds that correspond to the mean plus/minus one standard deviation of the 100 samples means). (A-C) Legends are the same as in Figure 7 in main text.

Figure S5

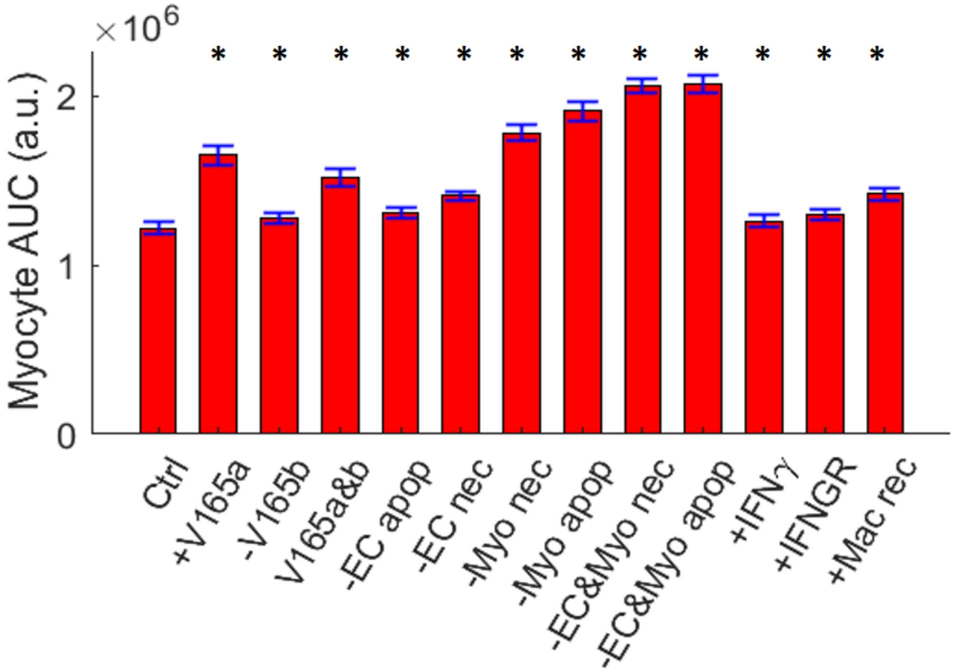

Figure S5. Simulated impact of targeted interventions on myocyte survival and regeneration under acute HLI in the virtual mouse population (VMP). Time-course integrals of myocyte population size in the VMP under control ('Ctrl') and a number of different targeted interventions under acute HLI. For labels, '+' indicates promotion, '-' indicates inhibition; abbreviations are V165a/b – $VEGF_{165}$a/b, apop – apoptotic death, nec – necrotic death, EC – endothelial cell, Myo – myocyte, IFNGR – interferon gamma receptor, Mac – macrophage, rec – recruitment. Details regarding model implementation of these interventions are available in Protocol S1. Integral values (in arbitrary units) computed from the 50 virtual mice are displayed as mean ± SEM (n=50); *$p<0.05$ compared to ctrl with a greater mean value; two-tailed paired t test was used.

Figure S6

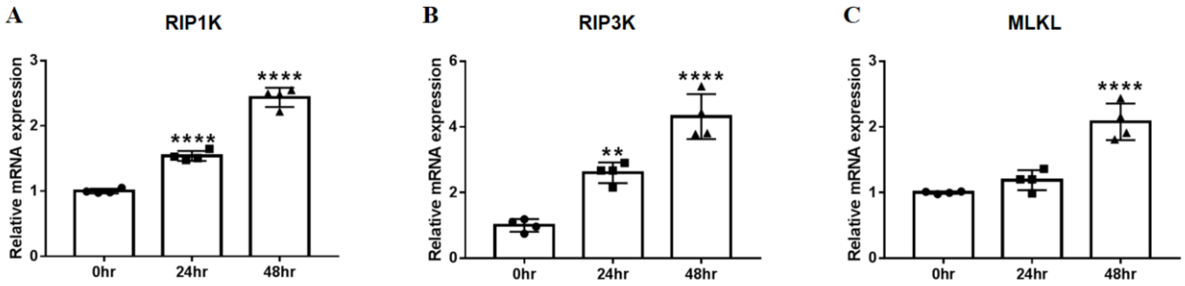

Figure S6. Necroptosis pathway expression is increased in endothelial cells exposed to hypoxia and serum starvation. Relative mRNA expression of (A) RIP1K (B) RIP3K and (C) MLKL in HUVECs following culture under hypoxia (2%) and serum starvation conditions (n=4). **p<0.01, ****p<0.001 compared to time 0 by one-way ANOVA followed by Tukey's post-hoc test for multiple comparisons. Data are mean±SEM.

Table S1

Part A

Reaction Flux & Rule Labels	Brief Reaction & Rule Description	Reaction Flux & Rule Details	Parameter Values Used in Reaction	References (e.g. compiled dose response data sources) for the estimated or optimized parameter values (in PMIDs); other parameters are fitted	Parameters and initial conditions (with the variation ranges) selected for generation of virtual mouse population
vt1	Recruitment and growth (simplified as one step) of myocytes	k1*(1-(IFNg/(IFNg+ka1)))*(1+1*((Vapcell*V165a_Availbility+Vbpcell)/((Vapcell*V165a_Availbility)+Vbpcell+kb1)))*(1+2*(HMGB1pcell/(HMGB1pcell+kc1)))*(1-((21/o2)^2/((21/o2)^2+kd1)))*(1+6*((Vapcell*V165a_Availbility+Vbpcell)/((Vapcell*V165a_Availbility)+Vbpcell+ke1)))	k1=3.564 (min^-1), ka1=15000, kb1=900000, kc1=30000, kd1=50, ke1=30000, V165a_Availability=0.5	ka1 (21576360), kb1 (dose response, 18094043, 15509502), kc1 (dose response, 17872450, 29203538), kd1 (dose response, 15798192), ke1 (dose response, 14507649)	ka1 (0.1x-10x), kb1 (0.1x-10x), kc1 (0.1x-100x), kd1 (0.2x-5x), V165a_Availability (IC: 0.2-0.8), ke1 (0.1x-10x)
vt2	Apoptosis of myocytes are induced by TNFa and hypoxia	k2*myo*(1+4*(TNFa^2/(TNFa^2+ka2)))*(1+3.5*((21/o2)^2/((21/o2)^2+kb2)))	k2=3e-5 (min^-1), ka2=8e8, kb2=400	k2 (14507649), ka2 (dose response, 18651291), kb2 (dose response, 31320610, 15262985, 28698543)	ka2 (0.1x-10x), kb2 (0.2x-5x)
vt3	Apoptosis of ECs are induced by TNFa, IFNg and hypoxia	k3*ec*(1+26*(TNFa/(TNFa+ka3)))*(1+4.5*(IFNg/(IFNg+kb3)))*(0.8+4.2*((21/o2)^2/((21/o2)^2+kc3)))	k3=3e-5 (min^-1), ka3=230000, kb3=5000, kc3=17.5	k3 (30118760,18618128), ka3 (dose response, 22038739), kb3 (dose response,	ka3 (0.1x-10x), kb3 (0.1x-10x), kc3 (0.2x-5x)

				24281189), kc3 (dose reponse, 25547648, 11463759, 10075703, 26459773, 24278276, 30118760, 11232639, 14633124)	
vt4	Production of V165a from myocytes and ECs	(ka4*ec+kb4*myo)*(1+3*((21/o2)^2/((21/o2)^2+kc4)))	ka4=1 (min^-1), kb4=1 (min^-1), kc4=100	ka4 (28656228), kc4 (dose response, 17132813)	kc4 (0.2x-5x), ka4 (0.5x-2x), kb4 (0.5x-2x)
vt5	Production of V165b from myocytes and ECs	ka5*ec+kb5*myo	ka5=1 (min^-1), kb5=1 (min^-1)	ka5 (assumed similar to ka4)	ka5 (0.5x-2x), kb5 (0.5x-2x)
vt6	Degradation of V165a in the tissue	k6*V165a	k6=0.008 (min^-1)	k6 (20642842,22104283)	
vt7	Degradation of V165b in the tissue	k7*V165b	k7=0.008 (min^-1)	k7 (assumed similar to k6)	
vt8	Proliferation of ECs (assuming logistic growth) are promoted by V165a, HMGB1 and inhibited by V165b	k8*ec*(1-(ec/ka8))*(1+(0.25+0.75*(Vapcell*V165a_Availbility/Vbpcell)^2/((Vapcell*V165a_Availbility/Vbpcell)^2+kb8))*(Vapcell*V165a_Availbility/Vbpcell)^2/((Vapcell*V165a_Availbility/Vbpcell)^2+kc8)*2*(Vapcell*V165a_Availbility/(Vapcell*V165a_Availbility+kd8)))*(1+0.7*(HMGB1pcell/(HMGB1pcell+ke8)))	k8=0.00105 (min^-1), ka8=3200, kb8=5, kc8=15, kd8=570, ke8=20000	kb8 (dose response, 12124351), kc8 (dose response, 20237252), ke8 (dose response, 25126750, 21944908)	kc8 (0.2x-5x), kb8 (0.2x-5x), kd8 (0.1x-10x), ke8 (0.1x-10x)
vt9	Tissue perfusion is influenced by	k9*perfusion*(ka9-perfusion)*(ec/ka8)*(1+kb9*mac	k9=7.5e-5 (min^-1), ka		

	the estimated degree of arteriogenesis (which is based on ECs, macrophages and the perfusion deficit) and the estimated degree of angiogenesis (which is based on ECs)	/(mac+kc9))+kd9*net_ecgrowth*perf_switch*(ka9-perfusion)	9=1, kb9=4, kc9=3500, kd9=4e-4		
vt10	Recruitment of macrophages into the tissue are promoted by CXCL9/10, HMGB1 and V165a but are inhibited by V165b	k10*(0.01+(CXCL10pcell+CXCL9pcell)/(CXCL10pcell+CXCL9pcell+ka10))*(1+((Vapmac*V165a_Availbility/Vbpmac)^2/((Vapmac*V165a_Availbility/Vbpmac)^2+kb10))*2.5*(Vapmac*V165a_Availbility/((Vapmac*V165a_Availbility)+kc10)))*(1+2.5*(HMGB1pcell/(HMGB1pcell+kd10)))	k10=8.47 (min^-1), ka10=600000, kb10=5, kc10=14000, kd10=2000	ka10 (dose response, 25561167), kc10 (dose response, 8605350, 29324807)	ka10 (0.1x-10x), kc10 (0.1x-10x), kb10 (0.2x-5x), kd10 (0.1x-10x)
vt11	Production of HMGB1 from necrotic cells	k12*(ka9-perfusion*nonleakiness)*myo*k11+k13*ec*(ka9-perfusion*nonleakiness)*k11	k11=1.2e6	k11 (23040637)	
vt12	Necrosis of myocytes depends on the deficit of tissue perfusion	k12*(ka9-perfusion*nonleakiness)*myo	k12=1e-4 (min^-1)		
vt13	Necrosis of ECs depends on the deficit of tissue perfusion	k13*ec*(ka9-perfusion*nonleakiness)	k13=0.00112 (min^-1)		
vt14	Removal of macrophages	k14*mac	k14=8.5e-4 (min^-1)		

	(all) from the tissue				
vt15	Degradation of HMGB1 in the tissue	k15*HMGB1	k15=0.04 (min^-1)	k15 (23447529)	
vt16	Removal of macrophages (the newest population) from the tissue	k14*mac	see vt14		
vt17	Recruitment of a new macrophage population into the tissue at each time step	k10*(0.01+(CXCL10pcell+CXCL9pcell)/(CXCL10pcell+CXCL9pcell+ka10))*(1+((Vapmac*V165a_Availbility/Vbpmac)^2/((Vapmac*V165a_Availbility/Vbpmac)^2+kb10))*2.5*(Vapmac*V165a_Availbility/((Vapmac*V165a_Availbility)+kc10)))*(1+2.5*(HMGB1pcell/(HMGB1pcell+kd10)))	see vt10		
vt18 (rule)	Assignment of tissue oxygenation based on perfusion and nonleakiness	o2 = 6*perfusion*nonleakiness			nonleakiness (IC: 0.5-1), perfusion (IC: 0-0.3 for acute HLI)
vt19 (rule)	Calculation of HMGB1 per cell	HMGB1pcell = HMGB1/(myo+ec+mac)*h1_anti			h1_anti (default value: 1)
vt20 (rule)	Calculation of V165a per cell	Vapcell = (V165a+Vamac)/(myo+ec)			
vt21 (rule)	Calculation of V165b per cell	Vbpcell = (V165b*vb_anti+Vbmac)/(myo+ec)			vb_anti (default value :1)

vt22 (rule)	Calculation of the net growth of EC	net_ecgrowth = k8*ec*(1-(ec/ka8))*(1+(0.25+0.75*(Vapcell*V165a_Availbility/Vbpcell)^2/((Vapcell*V165a_Availbility/Vbpcell)^2+kb8))*(Vapcell*V165a_Availbility/Vbpcell)^2/((Vapcell*V165a_Availbility/Vbpcell)^2+kc8)*2*(Vapcell*V165a_Availbility/(Vapcell*V165a_Availbility+kd8)))*(1+0.7*(HMGB1pcell/(HMGB1pcell+ke8)))-k3*ec*(1+26*(TNFa/(TNFa+ka3)))*(1+4.5*(IFNg/(IFNg+kb3)))*(0.8+4.2*((21/o2)^2/((21/o2)^2+kc3)))-k13*ec*(ka9-perfusion*nonleakiness)			
vt23 (rule)	Calculation of perf_switch	if net_ecgrowth>0, perf_switch=1; if net_ecgrowth<0, perf_switch=0			

Part B

Model Species Name	Initial Condition (copy numbers, rounded to the nearest integer)	Equations (dx/dt=, the 'v's correspond to the reaction fluxes in Part A); details of cell-level calculations are available in the sample MATLAB script provided	Dummy variable (e.g. placeholder)?
myo	90000	vt1-vt2-vt12	
ec	3000	vt8-vt3-vt13	
death	0	NA	Y
myo_rec	1	NA	Y

TNFa	0	assigned based on cell-level calculations	
V165a	3.75E+06	vt4-vt6	
V165b	3.75E+06	vt5-vt7	
deg	0	NA	Y
IFNg	0	assigned based on cell-level calculations	
EC_growth	1	NA	Y
perfusion	0.25 (for acute HLI), 1 (for normal)	vt9	
mac	500	vt10-vt14	
mac_rec	1	NA	Y
CXCL10pcell	0	assigned based on cell-level calculations	
CXCL9pcell	0	assigned based on cell-level calculations	
HMGB1	0	vt11-vt15	
nonleakiness	0.8 (for HLI), 1 (for normal)	NA	
V165a_Availability	0.5	NA	
o2 (percent)	6	see vt18	
Va_sec	1	NA	Y
Vb_sec	1	NA	Y
HMGB1pcell	0	see vt19	
Vapcell	0	see vt20	

Vbpcell	0	see vt21	
Vamac	0	assigned based on cell-level calculations	
Vbmac	0	assigned based on cell-level calculations	
mac_new	0	vt17-vt16	
Vapmac	0	assigned based on cell-level calculations	
Vbpmac	0	assigned based on cell-level calculations	
net_ecgrowth	0	see vt22	
perf_switch	0	see vt23	
h1_anti	1	NA	Y
necdeath	0	NA	Y
vb_anti	1	NA	Y

Table S1. Parts (A-B) Details of model reactions, initial conditions and parameter values of the tissue-level model component.

Table S2

Part A

Reaction Flux Labels	Brief Reaction Description (species are proteins unless otherwise noted)	Reaction Flux Details	Parameter Values Used in Reaction	References for Parameter Values (in PMIDs)
vm1	Binding of HMGB1 with TLR4	kf_vm1*[HMGB1]*[TLR4] - kr_vm1*[HMGB1/R]	kf_vm1=3.0e-8 (min^-1), kr_vm1=7 (min^-1)	30134799
vm2	TLR4 constitutive production	k_vm2	k_vm2=2.1 (min^-1)	Fitted
vm3	TLR4 constitutive degradation	k_vm3*[TLR4]	k_vm3=3.0e-4 (min^-1)	29449567
vm4	Internalization and shuttling of HMGB1/TLR4 complex	k_vm4*[HMGB1/R]	k_vm4=0.5 (min^-1)	Fitted
vm5	Degradation of internalized HMGB1/TLR4 complex	k_vm5*[HMGB1/R_lyse]	k_vm5=5.3 (min^-1)	Fitted
vm6	Recycling of TLR4	k_vm6*[HMGB1/R_lyse]	k_vm6=1.1 (min^-1)	Fitted
vm7	Translation of CXCL10	k_vm7*[mCXCL10]	k_vm7=6 (min^-1)	Fitted
vm8	Degradation of CXCL10	k_vm8*[C10]	k_vm8=0.002 (min^-1)	Fitted

vm9	Production of CXCL9	k_vm9*([pSTAT1D_n]^2)	k_vm9=3.0e-4 (min^-1)	Fitted
vm10	Degradation of CXCL9	k_vm10*[CXCL9]	k_vm10=0.002 (min^-1)	Fitted
vm11	IL-12 production is promoted by IRF1, IRF5, NFKB, CEBPB, AP1 and repressed by STAT3	k_vm11*([IRF1]^2/([IRF1]^2+ka46)+0.01)*(0.2+[aIRF5]/([aIRF5]+kb46))*(0.1+([NFKB_n]*[CEBPB]*[AP1_n]*0.0000001)/([NFKB_n]*[CEBPB]*[AP1_n]*0.0000001+kc46))*(1-[pSTAT3D_n]/([pSTAT3D_n]+kd46))	k_vm11=549 (min^-1), ka46=1e8, kb46=30000, kc46=100000, kd46=2	Fitted
vm12	Degradation of IL-12	k_vm12*[IL12]	k_vm12=0.001 (min^-1)	Fitted
vm13	PI3K phosphorylation is driven by receptor-ligand binding of IL-4, IL-10, VEGF165a, IL-1b, and HMGB1	k136*[PI3K]*([pIL4/R/JAK]+[pIL10/R/JAK]+[pV165a/R1]*0.033+[IL1b/R]*([IRAK4]+[pIRAK4])*3e-7+0.01+n_vm13*[HMGB1/R]/([HMGB1/R]+k_vm13))	k136=0.088 (min^-1), n_vm13=82, k_vm13=2000	Fitted
vm14	TRAF6 activation is promoted by IL-1b and HMGB1 signaling, and repressed by A20, miR-146b and SOCS1	k248*([HMGB1/R]*n_vm14+[IL1b/R]*([IRAK4]+[pIRAK4]))*[TRAF6]*(1.01-[A20]/([A20]+ka248))*(1-[SOCS1]/([SOCS1]+kb248))*(1.2-[m146b]/([m146b]+kc248))	k248=3.18e-7 (min^-1), ka248=300, kb248=100000, kc248=20, n_vm14=30000	Fitted

Note: Part A lists the newly added and modified parameters in the 8-pathway macrophage signaling component (compared to the previous version) included in our multiscale model. All other reaction fluxes, species values, equations and parameters are the same as in (28). Labels of parameters in Fig.6C (from bottom to top, see this part and also Table S1 in (28)): k4, kf3, kf2, kf1, k117, kr2, kr3, k134, kf14, kf205, kf199, kr11, k101, kf11, k12, k135, k209, k_vm7, k72, k204, k118, k_vm10, k_vm8, k95, k174.

Part B

Species Name	Initial condition (copy numbers, rounded to the nearest integer) and reference (if applicable)	Equations (dx/dt=, the 'v's correspond to the reaction fluxes in Part A)
HMGB1	0	-vm1
TLR4	7000, (PMID 25470552)	vm2-vm3-vm1+vm6
HMGB1/R	0	vm1-vm4
HMGB1/R_lyse	0	vm4-vm5-vm6
CXCL9	506, (PMID 25643352, 26892362)	vm9-vm10
C10 (CXCL10)	23600, (PMID 26892362, 19901067)	vm7-vm8

Note: Part B includes species initial conditions and equations for the newly added HMGB1/TLR4 axis; all other equations and species conditions are the same as in (28).

Table S2. Parts (A-B) Details of model reactions, initial conditions and parameter values of the cell-level (macrophage) model component.

Table S3

Fig #	Experimental Outcomes Measured (all results are normalized respectively)	Type of Assay/Experimental Technique	Cell Type (or other experimental condition)
2B	phosphorylated PI3K	western blot	Macrophages
2C	phosphorylated ERK	western blot	Macrophages
2D	TNFa secretion	ELISA	Macrophages
2E	IL-12 secretion	ELISA	Macrophages
2F	IL-10 secretion	ELISA	Macrophages
S1A	total IkB	western blot	Human Lung fibroblasts
S1B	phosphorylated JNK	western blot	Human Lung fibroblasts
S1C	phosphorylated P38	western blot	Human Lung fibroblasts
3A	Hindlimb Perfusion	Laser Doppler Perfusion Imaging	HLI
3B	Hindlimb muscle tissue oxygenation	TcPO2;Licox Clark-type oxygen and temperature probes	HLI
3C	Hindlimb muscle size	Muscle fiber cross section area	HLI
3D	EC cell population size in hindlimb tissue	flow cytometry	HLI
3E	Macrophage cell population size in hindlimb tissue	flow cytometry	HLI
3F	IFNg expression in hindlimb tissue	RT-PCR	HLI

3G	Arteriolar density in ischemic hindlimb	number of a-SM actin positive arterioles per muscle fiber (counted)	HLI
3H	Necrosis in hindlimb	Area of necrotic fiber by H&E staining	HLI
3I	Hindlimb Perfusion	Laser Doppler Perfusion Imaging	HLI
3J	Hindlimb muscle tissue oxygenation	Licox Clark-type oxygen and temperature probes	HLI
3K	Hindlimb muscle size	myofiber cross section area	HLI
3L	Macrophage cell population size in hindlimb tissue	Immunostaining	HLI

Table S3. Summary of literature data used in model calibration and validation.

References for Supplemental Materials

1. Higgins JP, Li T, Deeks JJ. Choosing effect measures and computing estimates of effect. Cochrane Handbook for Systematic Reviews of Interventions, 2019:143-176.
2. Hazarika S, Farber CR, Dokun AO et al. MicroRNA-93 controls perfusion recovery after hindlimb ischemia by modulating expression of multiple genes in the cell cycle pathway. Circulation 2013;127:1818-28.
3. Heuslein JL, McDonnell SP, Song J, Annex BH, Price RJ. MicroRNA-146a Regulates Perfusion Recovery in Response to Arterial Occlusion via Arteriogenesis. Front Bioeng Biotechnol 2018;6:1.
4. Ganta VC, Choi M, Kutateladze A, Annex BH. VEGF165b Modulates Endothelial VEGFR1-STAT3 Signaling Pathway and Angiogenesis in Human and Experimental Peripheral Arterial Disease. Circ Res 2017;120:282-295.
5. Meisner JK, Annex BH, Price RJ. Despite normal arteriogenic and angiogenic responses, hind limb perfusion recovery and necrotic and fibroadipose tissue clearance are impaired in matrix metalloproteinase 9-deficient mice. J Vasc Surg 2015;61:1583-94 e1-10.
6. Pellegrin M, Bouzourene K, Poitry-Yamate C et al. Experimental peripheral arterial disease: new insights into muscle glucose uptake, macrophage, and T-cell polarization during early and late stages. Physiol Rep 2014;2:e00234.
7. Shireman PK, Contreras-Shannon V, Ochoa O, Karia BP, Michalek JE, McManus LM. MCP-1 deficiency causes altered inflammation with impaired skeletal muscle regeneration. J Leukoc Biol 2007;81:775-85.
8. Jetten N, Donners MM, Wagenaar A et al. Local delivery of polarized macrophages improves reperfusion recovery in a mouse hind limb ischemia model. PLoS One 2013;8:e68811.
9. Beneke A, Guentsch A, Hillemann A, Zieseniss A, Swain L, Katschinski DM. Loss of PHD3 in myeloid cells dampens the inflammatory response and fibrosis after hind-limb ischemia. Cell Death Dis 2017;8:e2976.
10. Dokun AO, Keum S, Hazarika S et al. A quantitative trait locus (LSq-1) on mouse chromosome 7 is linked to the absence of tissue loss after surgical hindlimb ischemia. Circulation 2008;117:1207-15.
11. Tang GL, Chang DS, Sarkar R, Wang R, Messina LM. The effect of gradual or acute arterial occlusion on skeletal muscle blood flow, arteriogenesis, and inflammation in rat hindlimb ischemia. J Vasc Surg 2005;41:312-20.
12. McClung JM, McCord TJ, Southerland K et al. Subacute limb ischemia induces skeletal muscle injury in genetically susceptible mice independent of vascular density. J Vasc Surg 2016;64:1101-1111 e2.
13. Yang Y, Tang G, Yan J et al. Cellular and molecular mechanism regulating blood flow recovery in acute versus gradual femoral artery occlusion are distinct in the mouse. J Vasc Surg 2008;48:1546-58.
14. He M, Bianchi ME, Coleman TR, Tracey KJ, Al-Abed Y. Exploring the biological functional mechanism of the HMGB1/TLR4/MD-2 complex by surface plasmon resonance. Mol Med 2018;24:21.
15. Olea FD, Vera Janavel G, Cuniberti L et al. Repeated, but not single, VEGF gene transfer affords protection against ischemic muscle lesions in rabbits with hindlimb ischemia. Gene Ther 2009;16:716-23.
16. Muhlhauser J, Merrill MJ, Pili R et al. VEGF165 expressed by a replication-deficient recombinant adenovirus vector induces angiogenesis in vivo. Circ Res 1995;77:1077-86.
17. Waeckel L, Mallat Z, Potteaux S et al. Impairment in postischemic neovascularization in mice lacking the CXC chemokine receptor 3. Circ Res 2005;96:576-82.

18. Hawinkels LJ, Zuidwijk K, Verspaget HW et al. VEGF release by MMP-9 mediated heparan sulphate cleavage induces colorectal cancer angiogenesis. Eur J Cancer 2008;44:1904-13.
19. Artwohl M, Graier WF, Roden M et al. Diabetic LDL triggers apoptosis in vascular endothelial cells. Diabetes 2003;52:1240-7.
20. Cho DS, Schmitt RE, Dasgupta A, Ducharme AM, Doles JD. Single-cell deconstruction of post-sepsis skeletal muscle and adipose tissue microenvironments. J Cachexia Sarcopenia Muscle 2020;11:1351-1363.
21. Carreau A, El Hafny-Rahbi B, Matejuk A, Grillon C, Kieda C. Why is the partial oxygen pressure of human tissues a crucial parameter? Small molecules and hypoxia. J Cell Mol Med 2011;15:1239-53.
22. McKinley BA, Parmley CL, Butler BD. Skeletal muscle PO2, PCO2, and pH in hemorrhage, shock, and resuscitation in dogs. J Trauma 1998;44:119-27.
23. Gutierrez G, Lund N, Acero AL, Marini C. Relationship of venous PO2 to muscle PO2 during hypoxemia. J Appl Physiol (1985) 1989;67:1093-9.
24. Greenbaum AR, Etherington PJ, Manek S et al. Measurements of oxygenation and perfusion in skeletal muscle using multiple microelectrodes. J Muscle Res Cell Motil 1997;18:149-59.
25. Marino S, Hogue IB, Ray CJ, Kirschner DE. A methodology for performing global uncertainty and sensitivity analysis in systems biology. J Theor Biol 2008;254:178-96.
26. Lee CC, Wang CN, Lee YL, Tsai YR, Liu JJ. High mobility group box 1 induced human lung myofibroblasts differentiation and enhanced migration by activation of MMP-9. PLoS One 2015;10:e0116393.
27. Hattori Y, Enmi J, Iguchi S et al. Gradual Carotid Artery Stenosis in Mice Closely Replicates Hypoperfusive Vascular Dementia in Humans. J Am Heart Assoc 2016;5.
28. Zhao C, Medeiros TX, Sove RJ, Annex BH, Popel AS. A data-driven computational model enables integrative and mechanistic characterization of dynamic macrophage polarization. iScience 2021;24:102112.
